# Integrated Population Modeling Provides the First Empirical Estimates of Vital Rates and Abundance for Polar Bears in the Chukchi Sea

**DOI:** 10.1038/s41598-018-34824-7

**Published:** 2018-11-14

**Authors:** Eric V. Regehr, Nathan J. Hostetter, Ryan R. Wilson, Karyn D. Rode, Michelle St. Martin, Sarah J. Converse

**Affiliations:** 1 Marine Mammals Management, U.S. Fish and Wildlife Service, Anchorage, AK USA; 2 Present Address: Polar Science Center, University of Washington, Seattle, WA USA; 3 U.S. Geological Survey, Patuxent Wildlife Research Center, Laurel, MD USA; 4 U.S. Geological Survey, Alaska Science Center, Anchorage, AK USA; 5 U.S. Geological Survey, Washington Cooperative Fish and Wildlife Research Unit, School of Environmental and Forest Sciences (SEFS) & School of Aquatic and Fishery Sciences (SAFS), University of Washington, Seattle, WA USA

## Abstract

Large carnivores are imperiled globally, and characteristics making them vulnerable to extinction (e.g., low densities and expansive ranges) also make it difficult to estimate demographic parameters needed for management. Here we develop an integrated population model to analyze capture-recapture, radiotelemetry, and count data for the Chukchi Sea subpopulation of polar bears (*Ursus maritimus*), 2008–2016. Our model addressed several challenges in capture-recapture studies for polar bears by including a multievent structure reflecting location and life history states, while accommodating state uncertainty. Female breeding probability was 0.83 (95% credible interval [CRI] = 0.71–0.90), with litter sizes of 2.18 (95% CRI = 1.71–2.82) for age-zero and 1.61 (95% CRI = 1.46–1.80) for age-one cubs. Total adult survival was 0.90 (95% CRI = 0.86–0.92) for females and 0.89 (95% CRI = 0.83–0.93) for males. Spring on-ice densities west of Alaska were 0.0030 bears/km^2^ (95% CRI = 0.0016–0.0060), similar to 1980s-era density estimates although methodological differences complicate comparison. Abundance of the Chukchi Sea subpopulation, derived by extrapolating density from the study area using a spatially-explicit habitat metric, was 2,937 bears (95% CRI = 1,552–5,944). Our findings are consistent with other lines of evidence suggesting the Chukchi Sea subpopulation has been productive in recent years, although it is uncertain how long this will continue given sea-ice loss due to climate change.

## Introduction

Managing wildlife populations often requires knowledge of demographic parameters such as reproduction, survival, and density or abundance. This information is particularly important for species of conservation concern due to direct human impacts (e.g., harvest^1^, habitat fragmentation^2^) or changes in phenology, distribution, or population dynamics resulting from climate change^3^. Many species of large carnivores are imperiled^4^ and face accelerating declines^5^. Some of the characteristics that make these species vulnerable to extinction also make demographic parameters difficult to estimate, including low population densities, high mobility, expansive range requirements, and complex life histories. Resulting challenges include small sample sizes, heterogeneous detection probabilities^6^, non-random temporary emigration relative to a localized study area^7^, unobservable life history states^8^, and estimates of abundance that may not be referenced to the region of interest^9^. These issues can increase bias, reduce precision, and complicate interpretation of demographic parameters, limiting their value for management and conservation.

Polar bears (*Ursus maritimus*) occur at low densities throughout ice-covered waters of the Arctic (e.g., 0.0041 bears/km^2^)^10^ and typically have large annual activity areas (e.g., >100,000 km^2^)^11^. The global population of polar bears is divided into 19 subpopulations that currently exhibit variable demographic status, although up-to-date and accurate estimates of abundance and trend are often lacking^12^. In 2008, polar bears were listed as threatened range-wide under the U.S. Endangered Species Act (ESA) due to projected population declines associated with loss of sea-ice habitat resulting from climate change^13^. Estimates of demographic parameters are required for population projections^14^, compliance with protected species legislation (e.g., assessment of recovery criteria under the ESA)^15^, conservation assessments^16^, and management of subsistence harvest^17^. Given that habitat loss is projected to continue^18^, accurate and timely information will become increasingly important for state-dependent management (e.g., adjusting harvest based on current environmental conditions)^19^ and detection of potentially rapid or nonlinear population responses to climate change^20^.

The Chukchi Sea (CS) subpopulation of polar bears inhabits the Bering, Chukchi, and East Siberian seas^21^. Capture-recapture studies conducted 1986–1993 did not provide adequate information to estimate demographic parameters due to low recapture rates and the movement of bears in to and out of the study area^22^. The only previous estimate of abundance was based on a rough extrapolation of the number of maternity dens on Wrangel Island, an important denning area located north of mainland Russia, in the 1970s and 1980s^23^. Although recent research has suggested positive nutritional condition and reproduction despite sea-ice loss^24,25^, abundance has never been estimated using empirical methods with a clear spatial and temporal reference, and the IUCN Polar Bear Specialist Group lists abundance and trend of the CS subpopulation as unknown^26^.

In this study, we develop an integrated population model (IPM)^27^ to analyze several types of count data together with multistate capture-recapture and telemetry data that include uncertainty in the true state (e.g., reproductive class) of individuals^28^. After fitting the model, we use estimates of abundance referenced to the study area, together with polar bear movement data and a habitat-quality metric^29^, to extrapolate density throughout the subpopulation range^30^. Our objectives were to (i) estimate abundance for the CS subpopulation using data collected in a geographically-limited study area; (ii) estimate vital rates (e.g., reproduction and survival) by concurrently analyzing multiple types of data, including parameters that would be unobservable or confounded if data types were analyzed separately; (iii) reduce bias in demographic parameters by modeling the movement of animals in to and out of the study area^31,32^; and (iv) develop a flexible modeling framework that addresses key analytical challenges for CS polar bears, and can be adapted to other subpopulations or species. Our findings represent the first rigorous estimates of demographic parameters for the CS polar bear subpopulation, providing information needed for management and serving as a baseline to evaluate future population change.

## Methods

### Study Area and Data Collection

**Figure 1:**
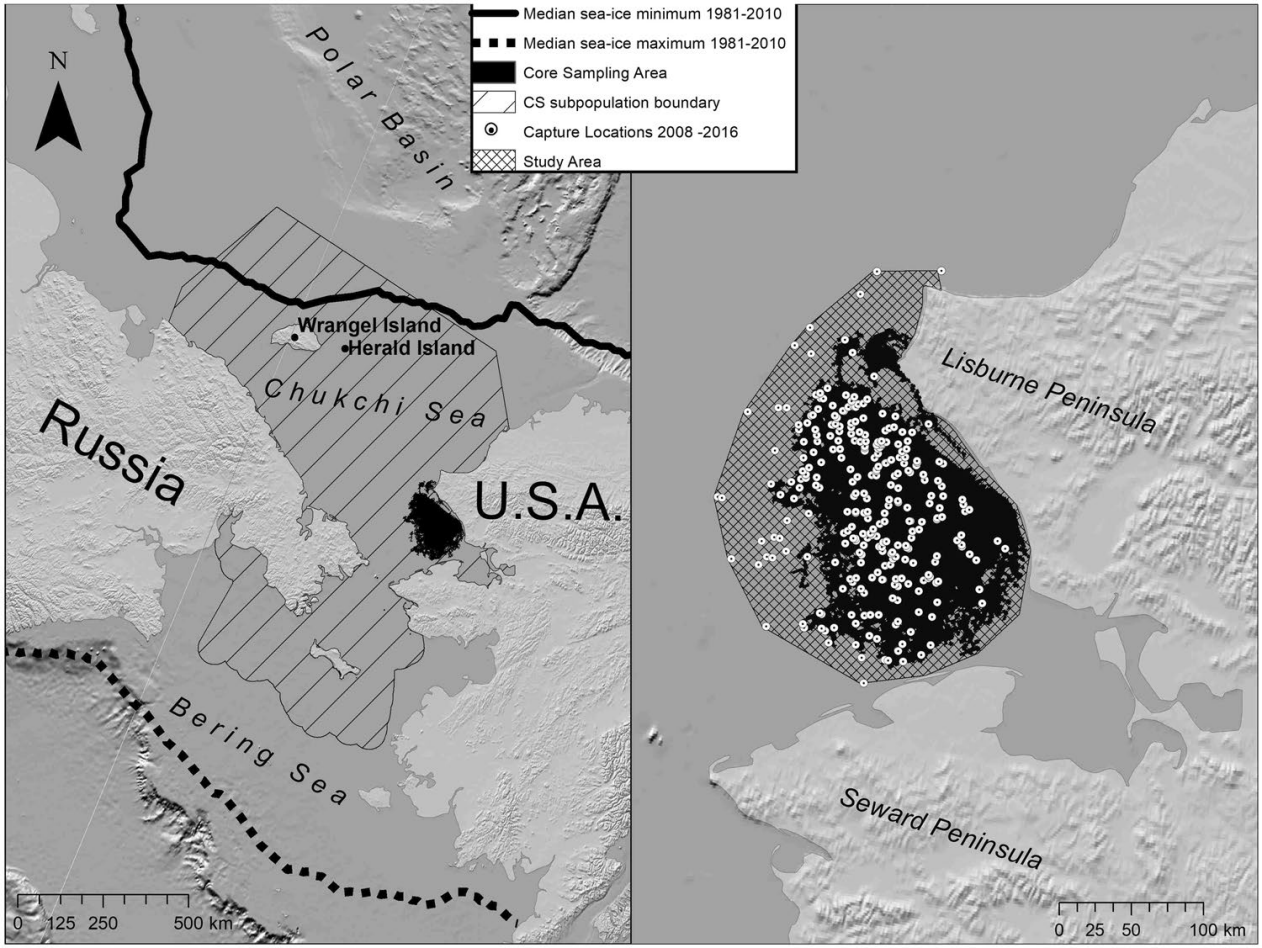
Location of the Chukchi Sea (CS) polar bear subpopulation. Polar bears were captured on the sea ice in the study area located west of Alaska, 2008–2016. Minimum and maximum sea-ice extent were obtained from Fetterer *et al*.^76^.

The CS region is seasonally covered by sea ice that extends south into the Bering Sea at its maximum extent in March and retreats north towards the polar basin at its minimum extent in September^33^. We physically captured polar bears in an offshore study area located west of Alaska (Fig. 1) from mid-March to early May in 2008–2011, 2013, and 2015–2016, using standard chemical immobilization techniques^34^. For use in some analyses, we defined a multiyear core sampling area of approximately 25,000 km^2^ based on Global Positioning System (GPS) track log locations for the helicopter used in captures (Fig. 1). Dependent young (age-zero cubs [C0], age-one cubs [C1], and age-two cubs [C2] still accompanying their mother) were aged based on body size and dentition. Most captured females age ≥4 years were fitted with satellite collars (2008–2010; Telonics, Mesa, AZ, USA) or GPS collars (2011–2016; Telonics).

Physical captures resulted in four primary data types used in analyses: (1) annual counts of bears of known sex and age, (2) annual counts of C1 litter sizes, (3) annual counts of dependent (i.e., still with their mothers) versus independent C2s, and (4) capture-recapture and telemetry data to inform the life-cycle state of individually-identified bears. Whereas physical captures occurred within the study area only, telemetry data were analyzed to remotely obtain information on collared bears (e.g., their location and reproductive status) and to evaluate springtime use areas (Supplementary Methods).

Estimates of abundance in this paper are referenced to the CS subpopulation boundary, as recognized by the IUCN Polar Bear Specialist Group^26^, with the southern boundary modified to exclude regions that were not confirmed to have been used by polar bears based on telemetry data in this study. Additional details about the study area and data collection are provided in the Supplementary Methods.

### Integrated Population Model

We built an IPM to estimate vital rates and abundance. Most model parameters were time constant due to small sample sizes, and were defined relative to biologically relevant sex, age, and reproductive classes as established by other polar bear studies^14,35^. A general description of the model is provided here, with additional details in the Supplementary Methods. The IPM is graphically displayed in Supplementary Fig. S1. A complete list of parameter, data, and indexing definitions is provided in Supplementary Table S1.

#### Projection matrix

**Figure 2:**
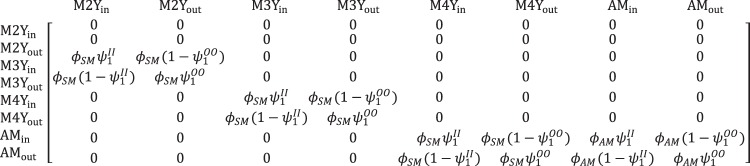
Male life cycle matrix in the integrated population model for polar bears. Rows and columns represent life cycle states. The parameters in cell *i*,*j* define the probability of transitioning from the state in column *j* at year *t*, to the state in row *i* at year *t* + 1. Life cycle states and parameters are described in the main text and Supplementary Table S1. Superscripts *I* and *O* on movement probabilities (*ψ*) designate states that are inside and outside the study area, respectively. Male recruitment (not shown) matches female recruitment (Fig. 3) assuming an equal sex ratio.

**Figure 3:**
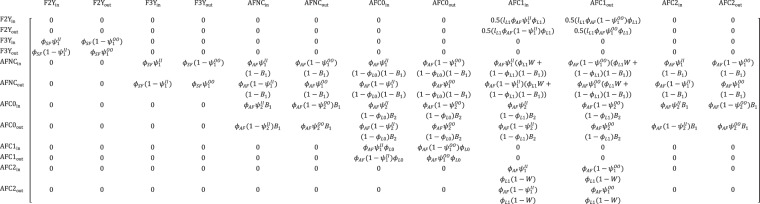
Female life cycle matrix in the integrated population model for polar bears in the Chukchi Sea. Rows and columns represent life cycle states. The parameters in cell *i*,*j* define the probability of transitioning from the state in column *j* at year *t*, to the state in row *i* at year *t* + 1. Life cycle states and parameters are described in the main text and Supplementary Table S1. Superscripts *I* and *O* on movement probabilities (*ψ*) designate states that are inside and outside the study area, respectively. In addition, but not shown, is a single absorbing dead state.

The IPM is based on a matrix projection model that reflected the polar bear life cycle^19^ and included states (*s* = 1, 2, … *S*) representing sex, age, reproductive status, and location (i.e., whether an animal was in or out of the study area; Figs 2 and 3). To account for demographic stochasticity we modeled population processes as binomial and multinomial outcomes, as described below. Recruitment was determined by reproductive parameters in the female model, with bears entering the male and female projection matrices at age 2 years with an equal sex ratio (Figs 2 and 3).

The male population projection model included four age-classes representing annual ages for subadults (2–4 years; states M2Y, M3Y, and M4Y) and adults (≥5 years; state AM; Fig. 2)^35^. The model allowed for temporary emigration by including versions of each state that were in and out of the study area. Transitions among states were determined by subadult survival (*ϕ*_*SM*_), adult survival (*ϕ*_*AM*_), and movement probabilities that allowed individuals outside of the study area in year *t* to either remain outside (${\psi }_{1}^{OO}$) or move into the study area ($1-{\psi }_{1}^{OO}$) in year *t* + 1. Similarly, individuals inside the study area could remain inside (${\psi }_{1}^{II}$) or move outside ($1-{\psi }_{1}^{II}$). We did not enforce any relationship between these parameters, thereby allowing for Markovian movements.

For females, the population projection model included states representing annual age classes for subadults (2–3 years; states F2Y and F3Y), after which bears were considered adults (≥4 years). Adult females and their dependent young were represented by four states: adult females with no cubs (AFNC), age-zero cubs (AFC0), age-one cubs (AFC1), and age-two cubs (AFC2; Fig. 3). Adult females with C0s were rare in the study area because most females give birth in maternal dens on land in Russia, and post-denning land departure occurs in late March^36^ making them unavailable for capture. Transitions among adult female states were determined by survival, movement, breeding, weaning, and litter survival probabilities (i.e., the probability that at least one cub in a litter survives until the following year; Fig. 3). Similar to males, we specified separate subadult and adult survival probabilities for females (*ϕ*_*SF*_ and *ϕ*_*AF*_, respectively), and female age- and reproductive-classes could be in or out of the study area (Fig. 3). Females entering state AFC0 were allowed different movement probabilities (${\psi }_{2}^{II},{\psi }_{2}^{OO}$) than other bears, because parturient females were more likely to move out, or remain out, of the study area due to selection for denning habitats in Russia.

We defined breeding for a female polar bear as consisting of fertilization in the spring of year *t*, implanting the blastocyst and entering a maternal den in autumn or winter, and emerging with at least one C0 in the spring of year *t* + 1^37^. We included two female breeding probabilities: *B*_1_ for females without cubs or with C2 (i.e., females that are available to breed per a typical three-year reproductive cycle), and *B*_2_ for females with C0 or C1. We expected *B*_2_ < *B*_1_, because *B*_2_ requires that a female lose her entire C0 or C1 litter in time to breed again in the same spring^35^.

#### Data

For state-specific count data, we modeled the number of individuals physically captured in state *s* and year *t* (*n*_*s*,*t*_) as binomially distributed random variables, with capture probability *p* and latent state-specific abundance *N*_*s*,*t*_:

1 $${n}_{s,t} \sim Binomial\,(p{I}_{s},{N}_{s,t}),$$

where *I*_*s*_ is an indicator variable equal to 1 if state *s* is an inside state (i.e., inside the study area) and 0 otherwise. Capture probability was constant across states and years except that it was fixed to 0 in 2012 and 2014 when there was no sampling.

We modeled annual counts of C1 litter sizes (*n*_*L*1[1:3],*t*_), observed during physical captures, as multinomial random variables. Parameters of the multinomial (*ω*_*L*1[1:3]_) described the probability that an AFC1 has 1, 2, or 3 age-one cubs^37^. Following the methods of Hunter *et al*.^38^ and Regehr *et al*.^19^, the parameters *ω*_*L*1[1:3]_ were modeled as a function of C0 litter size probabilities (*ω*_*L*0[1:3]_) and C0 survival (*ϕ*_*C*0_). These multinomial probabilities were modeled as conditional on at least one C0 surviving (*ϕ*_*L*0_; Supplementary Methods). That is, if at least one cub in a female’s C0 litter was predicted to have survived, we modeled the probability that she had 1, 2, or 3 C1s. Expected C0 and C1 litter sizes (*l*_*L*0_ and *l*_*L*1_, respectively) were then derived parameters. The number of dependent and independent C2s provided information on the probability (*W*) that C2s were weaned (i.e., separated from their mothers) prior to the spring sampling period. Here, we assumed:

2 $${n}_{weanC2,t} \sim Binomial\,(W,{n}_{C2,t}),$$

where *n*_*weanC*2,*t*_ is the number of observed independent C2 cubs in year *t*, *n*_*C*2,*t*_ is the total number of observed C2 cubs, and *W* is the probability that a C2 weaned prior to our sampling period.

Capture-recapture and telemetry data were jointly analyzed using a multievent model^39^ with true latent states matching those defined by the population projection matrices, excluding the recruitment component. Conditional on first capture, we assumed the state of an individual in year *t* was a categorical random variable:

3 $${z}_{i,t}|{z}_{i,t-1} \sim Cat({{\rm{\Theta }}}_{{z}_{i,t-1}}),$$

where ${{\rm{\Theta }}}_{{z}_{i,t-1}}$ is the vector of state transition probabilities for an individual that was in state *z*_*i*,*t*−1_ in year *t*−1, as defined by parameters in the projection matrices (Figs 2 and 3).

We modeled observation data for individual *i* in year *t*, *y*_*i*,*t*_, as a function of its state in year *t* as well as individual- and time-specific factors (e.g., presence of a functional collar). We assumed *y*_*i*,*t*_ is a categorical random variable:

4 $${y}_{i,t}|{z}_{i,t} \sim Cat({{\rm{\Pi }}}_{i,t,{z}_{i,t}}),$$

where ${{\rm{\Pi }}}_{i,t,{z}_{i,t}}$ is the vector of detection probabilities for individual *i* in year *t*. For males, only direct observation data were available, thus individuals inside the study area were detected with probability *p*, and individuals outside the study area were detected with zero probability (i.e., could not be observed). For females, the observation process included both direct and telemetry observations, where the true state may be partially identified from remote telemetry observations (e.g., a collared individual was known to be alive and outside the study area, but its reproductive status was uncertain). Details of the observation component of the IPM are provided in the Supplementary Methods.

We calculated the total number of individuals that used the study area in year *t* (*N*_*study*,*t*_) as the sum of the number of bears in all inside states, plus the estimated number of dependent cubs associated with adult females:

5 $${N}_{study,t}=\sum _{s[{\rm{in}}]}\,{N}_{s,t}+{N}_{C0[{\rm{in}}],t}+{N}_{C1[{\rm{in}}],t}$$

where *s*[in] denotes all inside states. Annual abundances of C0s (*N*_*C*0[in],*t*_) and C1s (*N*_*C*1[in],*t*_), which were always with their mothers and therefore not included as independent individuals, were calculated as the product of average C0 and C1 litter sizes and the total (latent) number of females in each reproductive state inside the study area (*N*_*AFC*0[in],*t*_ and *N*_*AFC*1[in],*t*_, respectively). A multiyear average study area abundance (${\bar{N}}_{study}$) was calculated within the IPM as the mean of annual estimates. Details of abundance estimation are provided in the Supplementary Methods.

### Model Implementation

To fit the IPM, we used informative Beta priors for subadult and adult survival, corresponding to mean values and standard deviations on the probability scale of 0.89 (sd = 0.05), 0.93 (sd = 0.02), 0.82 (sd = 0.10), and 0.89 (sd = 0.05) for *ϕ*_*SF*_, *ϕ*_*AF*_, *ϕ*_*SM*_, and *ϕ*_*AM*_, respectively. These priors were developed using moment matching methods based on point estimates of total survival (i.e., including harvest mortality) from capture-recapture studies for 12 polar bear subpopulations with available data (Supplementary Methods, Supplementary Table S3). We used an informative Beta(2.1,11.4) prior for *B*_2_^35^ as it was only weakly identifiable from the data. Vague priors were used for all other parameters. We fit the model in a Bayesian framework using JAGS^40^ and the *jagsUI* package^41^ accessed through R version 3.3.1 (R Core Team 2016). Further details about priors, sensitivity of estimated parameters to choice of priors, model goodness-of-fit, and implementing the IPM are provided in Supplementary Methods. We report results as posterior modes and 2.5^th^ and 97.5^th^ quantiles unless otherwise noted.

### Density Extrapolation

After fitting the model, we used a previously developed, spatially- and temporally-explicit habitat-quality metric^29^ to extrapolate density estimates from the study area to the CS subpopulation boundary. First, we estimated a multiyear, average density within the core sampling area, after correcting for lack of geographic closure (${\bar{D}}_{sampling}$), as follows:

6 $${\bar{D}}_{sampling}={\bar{N}}_{study}\times \hat{q}/{A}_{sampling},$$

where $\hat{q}$ is the average proportion of the individual areas used by collared females during the spring sampling season (Supplementary Methods) that occurred within the core sampling area; and *A*_*sampling*_ is the size (km^2^) of the core sampling area^42^. Second, we calculated an adjusted value of local density that excluded AFC0 and C0 (${\bar{D}}_{sampling}^{\ast }$,), by replacing ${\bar{N}}_{study}$ in equation (6) with a multiyear average abundance estimate that excluded *N*_*AFC*0,*t*_ and *N*_*C*0,*t*_. Because AFC0 and C0 rarely used the study area in the spring (i.e., there were only 3 direct observations of AFC0), we used ${\bar{D}}_{sampling}^{\ast }$ to extrapolate densities of bears in other states, and subsequently added in approximate numbers of AFC0 and C0 (see below; Supplementary Methods). Third, we overlaid the region with 25 × 25 km grid cells. For each grid cell *x*, we used a habitat-quality metric (*h*_*x*_) representing the relative probability of use by polar bears during March and April, averaged over the years 2009–2011, 2013, and 2015–2016, as estimated from non-denning adult female polar bear location data and environmental covariates (e.g., sea-ice concentration and characteristics, ocean depth) using resource selection functions^29^. Fourth, we extrapolated abundance to the entire CS subpopulation boundary area (${\bar{N}}_{CS}^{\ast }$) by assuming a 1:1 proportional relationship between habitat quality and density^30^, as follows:

7 $${\bar{N}}_{CS}^{\ast }=({\bar{D}}_{sampling}^{\ast }\times \,{A}_{sampling})\times \sum _{x=1}^{{X}_{CS}}\,{h}_{x}/\sum _{x=1}^{{X}_{sampling}}\,{h}_{x},$$

where ${\bar{N}}_{CS}^{\ast }$ is an extrapolated estimate of abundance, excluding AFC0 and C0, referenced to the CS subpopulation boundary; and *X*_*sampling*_ and *X*_*CS*_ are the number of grid cells overlaying the core sampling area and CS subpopulation boundary, respectively. We used bootstrapping methods to estimate variance and account for uncertainty in the proportions of AFC0 and C0 that occurred within the total subpopulation (Supplementary Methods).

### Age-One Cubs per Adult Female

Separate from other analyses, we used the physical capture data to estimate and evaluate temporal trends during the period 2008–2016 in the number of C1s per adult female, a metric that integrates cub production and first-year survival^15^. We assumed the annual numbers of C1s were Poisson distributed random variables with an offset for the numbers of adult females, and compared the fit of constant and linear trend models. The models were fit in JAGS following the same specifications as the IPM.

### Ethics statement

This research was approved by and carried out in accordance with (i) the U.S. Marine Mammal Protection Act and ESA, under U.S. Fish and Wildlife Service (USFWS) permit number MA046081; and (ii) animal handling protocols established by the USFWS Region 7 Institutional Animal Care and Use Committee.

## Results

A total of 166 unique males (annual mean = 24, sd = 5) and 135 unique females (annual mean = 19 bears, sd = 5 bears) were physically captured and released in 2008–2011, 2013, and 2015–2016. These numbers do not reflect captures of C0s and C1s, which were not included as individuals in the capture-recapture model. Among adult females, 103 individuals (annual mean = 15, sd = 3) received telemetry collars. The complete dataset consisted of 403 direct and remote observation events (Supplementary Table S2), 39 observations of C1 litters (Supplementary Table S4), and 61 observations of independent and dependent C2s (Supplementary Table S5).

The separate analysis of the number of C1s per adult female provided no evidence for a linear trend during the period 2008–2016 (slope = 0.02, 95% credible interval = −0.07–0.10). The time-constant model resulted in an estimate of 0.64 (0.49–0.80) C1s per adult female.

### Vital Rates and Movement Probabilities

**Figure 4:**
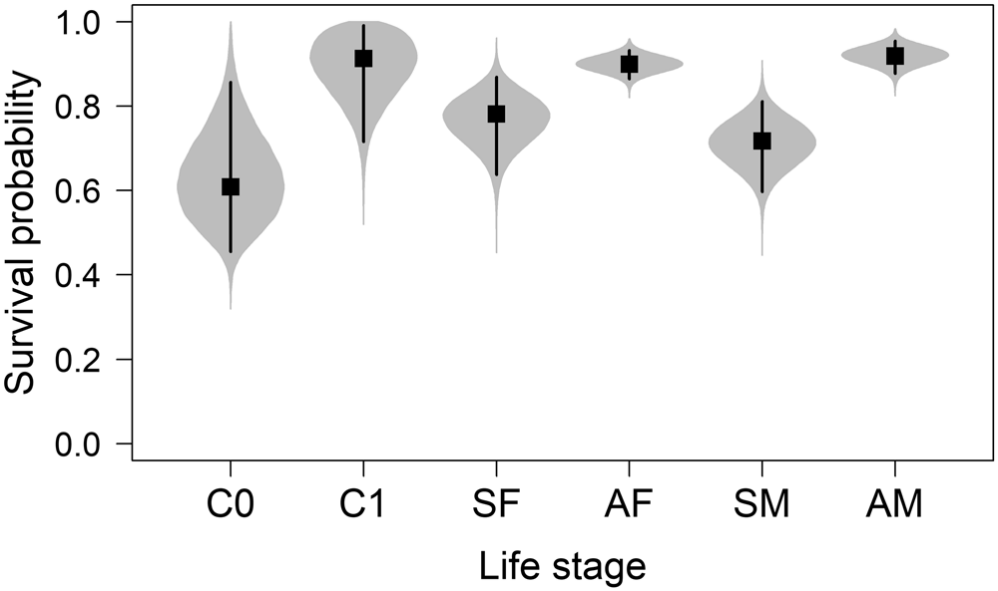
Apparent survival of polar bears in the Chukchi Sea. Life stages are age-zero cubs (C0), age-one cubs (C1), subadult females (SF), adult females (AF), subadult males (SM), and adult males (AM). Posterior distributions (violin plots), modes (squares), and 95% credible intervals (error bars) are shown. Note that cub survival is conditional on adult female survival (i.e., survival of the mother).

Survival probability modes varied by sex and age, with overlapping credible intervals (Fig. 4). Adult female and male survival probabilities were 0.90 (0.86–0.92) and 0.89 (0.83–0.93), respectively (Table 1). Subadult female and male survival were 0.79 (0.68–0.87) and 0.71 (0.59–0.81), respectively. Age-zero cub survival was 0.62 (0.45–0.86). Litter survival probabilities for C0 (*ϕ*_*L*0_) and C1 (*ϕ*_*L*1_) were 0.87 (0.82–1.00) and 0.96 (0.83–1.0), respectively.

**Table 1:** Vital rates for polar bears in the Chukchi Sea.

Parameter	Mode (95% CRI)
*ϕ*_*C*0_	0.62 (0.45–0.86)
*ϕ*_*C*1_	0.92 (0.71–0.99)
*ϕ*_*SF*_	0.79 (0.68–0.87)
*ϕ*_*AF*_	0.90 (0.86–0.92)
*ϕ*_*SM*_	0.71 (0.59–0.81)
*ϕ*_*AM*_	0.89 (0.83–0.93)
${\psi }_{1}^{II}$	0.59 (0.40–0.73)
${\psi }_{1}^{OO}$	0.78 (0.64–0.89)
${\psi }_{2}^{II}$	0.01 (0.00–0.12)
${\psi }_{2}^{OO}$	0.98 (0.89–0.99)
*B*_1_	0.83 (0.71–0.90)
*B*_2_	0.10 (0.02–0.39)
*W*	0.34 (0.24–0.44)
*l*_*L*0_	2.18 (1.71–2.82)
*l*_*L*1_	1.61 (1.46–1.80)

Values are posterior modes and 95% credible intervals (CRI). Parameters included survival probabilities (*ϕ*), movement probabilities (*ψ*), breeding probabilities (*B*), weaning probability (*W*), and average litter size (*l*). Detailed parameter definitions are provided in the main text and Supplementary Table S1.

Breeding probability for states AFNC and AFC2 (*B*_1_) was 0.83 (0.71–0.90; Table 1). As expected, this was higher than breeding probability for states AFC0 and AFC1 (*B*_2_ = 0.10; 0.02–0.39). The average size of C0 and C1 litters was 2.18 (1.71–2.82) and 1.61 (1.46–1.80) individuals, respectively. The probability that a C1 in year *t* would be weaned prior to the sampling period in year *t* + 1 (*W*) was 0.34 (0.24–0.44).

Denning females were generally outside the study area during the sampling period, as indicated from previous studies of reproductive ecology in this subpopulation^36^. Specifically, females entering state AFC0 moved out of the study area ($1-{\psi }_{2}^{II}$) with probability 0.99 (0.88–1.00) or remained outside (${\psi }_{2}^{OO}$) with probability 0.98 (0.89–0.99; Table 1). Bears not transitioning into state AFC0 exhibited a tendency to stay where they were the previous year, either remaining in the study area (${\psi }_{1}^{II}$) with probability 0.59 (0.40–0.73) or remaining outside the study area (${\psi }_{1}^{OO}$) with probability 0.78 (0.64–0.89). Estimated detection probabilities and assignment parameters for the multievent capture-recapture data are provided in the Supplementary Results.

### Density and Abundance

**Figure 5:**
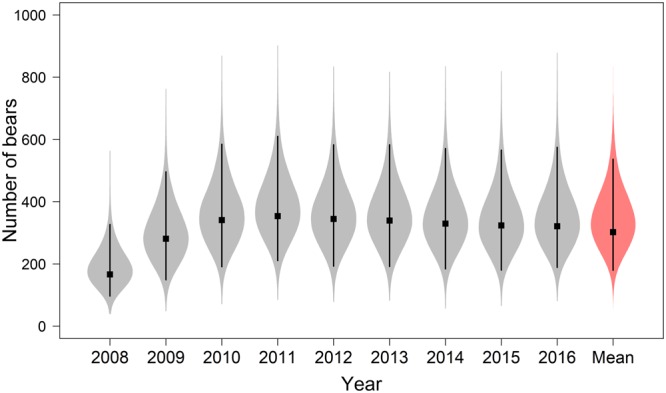
Annual estimates of the number of Chukchi Sea polar bears that used the study area during the sampling period. Violin plots represent the full posterior distributions, squares are modes, and error bars are 95% credible intervals. The multiyear mean estimate is shown in red.

Annual estimates of the number of bears that used the study area (*N*_*study*,*t*_) ranged from 167 (94–312) in 2008 to 350 (208–589) in 2011, with a multiyear mean ${\bar{N}}_{study}$ = 296 (176–513; Fig. 5). The average proportion of time that collared adult females spent inside the core sampling area, over the approximate 6-week duration of the sampling period, was $\hat{q}$ = 0.25 (0.16–0.36). Multiyear average density within the core sampling area was ${\bar{D}}_{sampling}$ = 0.0030 bears/km^2^ (0.0016–0.0060). The multiyear average estimate of abundance, extrapolated to the area within the CS subpopulation boundary and adjusted to include approximate numbers of AFC0 and C0, was ${\bar{N}}_{CS}$ = 2,937 (1,552–5,944). This corresponds to an average density of 0.0036 bears/km^2^ (0.0019–0.0073) within the CS subpopulation boundary.

## Discussion

Many large carnivores have experienced range retractions, are threatened with extinction, or both. Factors that contribute to vulnerability include intrinsic biological traits (e.g., complex life histories with extended maternal care) and characteristics that increase contact with humans (e.g., high mobility and expansive range requirements), both of which can pose challenges for research. In this paper we develop an IPM that combines multiple data types into a single, integrated analysis of abundance and demographic processes^43^. We used telemetry data to model animal movements in to and out of the study area, with a multievent structure^28^ to account for state uncertainty. After fitting the IPM, we used a habitat-quality metric calculated from resource selection functions to extrapolate densities. This allowed large-scale inference from geographically-limited sampling^44^, producing estimates of abundance with a clear spatial and temporal definition (i.e., as opposed to estimates of “superpopulation” size from open capture-recapture models, which can be difficult to interpret)^9^.

### Vital Rates

Survival rates for independent bears in this study represented total apparent survival (i.e., the probability of remaining alive, considering all sources of mortality, and not permanently emigrating from the study area). We did not estimate harvest mortality because only five animals were captured as independent bears and subsequently known to be killed by humans, which was too few to justify additional model complexity. Based on an average harvest from 2008–2015 of approximately 24 bears per year in Alaska^45^ and 32 bears per year in Chukotka^46^, and a total abundance of 2,937 bears (this study), the median proportion of the CS subpopulation removed by humans each year would be approximately 1.8% (0.9–3.6%). This is likely below the 4.5% harvest rate, at a 2:1 male-to-female harvest sex ratio, that has been commonly applied to polar bear subpopulations under favorable environmental conditions^19^, noting that 1.8% likely represents a minimum estimate due to incomplete harvest reporting^45,46^.

We used telemetry data to model animal movements and thereby mitigate potential bias in demographic parameters due to non-random temporary emigration^7^. We found that the probability of being in the study area in year *t* differed for bears that were in vs. out in year *t* – 1, and was a function of reproductive state (i.e., adult females with C0s had very low probability of being in). In a review of the demographic status of 13 Canadian subpopulations, York *et al*.^32^ suggested that incomplete geographic sampling was significantly correlated with negative bias in demographic parameters. Furthermore, a data-based simulation study for the Southern Beaufort Sea subpopulation identified non-random temporary emigration and movement-related heterogeneity in recapture probability as key factors contributing to bias when the study area is smaller than the subpopulation range^31^. Although we took an important step toward addressing this challenge, estimates of survival for independent bears in this study should be interpreted with caution. Recapture probabilities (*p*) were conditional on presence in the study area, as determined by movement probabilities (*ψ*). Due to sample size limitations, estimates of both *p* and *ψ* were restricted to be constant across time and multiple life cycle states. Also, estimates of *ψ* were informed primarily by data on adult females because other sex and age classes did not provide telemetry data, which contributed more to estimation of movements than did direct captures. Polar bears are mobile animals that exhibit individual and interannual variation in site fidelity and activity area sizes^11^. Although net movement patterns appear broadly similar for adult males and females^47^, seasonal movements may reflect sex-specific behavioral differences^48^. These factors likely introduced un-modeled heterogeneity in *p*, which can negatively bias survival estimates^6^. Also, heterogeneity in *ψ* can confound temporary and permanent emigration. Our core sampling area comprised only ~3% of the total area within the CS subpopulation boundary. Thus, some individuals likely had zero probability of returning following initial capture, a phenomenon that would be indistinguishable from mortality.

Estimates of reproductive parameters provide context for the current status of the CS subpopulation. Relative to 12 polar bear subpopulations with available data as summarized in Regehr *et al*.^19^, our estimates of breeding probability and age-zero cub (C0) survival were average (*B*_1_ = 0.83 compared to mean point estimate for bears age ≥6 years = 0.81, range = 0.44–1.0; and *ϕ*_*C*0_ = 0.62 compared to mean point estimate = 0.63, range = 0.34–0.90, respectively), and our estimates of C0 litter size and age-one cub (C1) survival were high (*l*_*L*0_ = 2.16 compared to mean point estimate = 1.64, range = 1.49–1.76; and *ϕ*_*C*1_ = 0.92 compared to mean point estimate = 0.81, range = 0.32–0.98, respectively). Detailed comparisons are complicated by methodological differences and lack of information on subpopulation sizes relative to environmental carrying capacity (*K*), which determines the strength of density-dependent regulation. It is possible that the CS subpopulation size is currently above its maximum net productivity level, which Regehr *et al*.^19^ suggested occurs at approximately 0.69*K* for polar bears, due to (i) relatively low harvest in recent years, and (ii) loss of approximately 8.9 ice-covered days per year for the period 1979–2014^49^, which is likely correlated with reduced on-ice foraging opportunities for polar bears^36^ and may cause reductions in *K* or intrinsic growth rate. However, it is unknown whether unquantified, but likely high, illegal harvest in Russia during the period 1994–2003^46^ caused reductions from which the CS subpopulation is still recovering.

Our findings of average-to-high reproductive parameters appear consistent with ecological research indicating that body condition and reproduction of CS bears did not decline between 1986–1994 and 2008–2011^24^. For 2008–2011, these metrics were also higher for the CS subpopulation compared to the adjacent Southern Beaufort Sea subpopulation^24^, which is experiencing negative demographic effects of sea-ice loss^35,50^. Possible explanations for the productivity of CS bears include high biological productivity in the region^51^, the positive status of ice-seal populations^52^, and a short history of years in which the sea ice melts completely over the continental shelf^24^. In this study, we found that the number of C1s per adult female did not exhibit a linear trend during the period 2008–2016, and was similar to a previous estimate for the period 1986–1994 (0.64 and 0.62, respectively^24^). This suggests that productivity of CS bears has not declined in recent decades. Due to sample size limitations, within the IPM framework we were not able to evaluate temporal variation in vital rates or relationships between vital rates and environmental conditions (e.g., sea-ice availability), which have been detected for some polar bear subpopulations^14,50^ and are important for understanding demographic status relative to climate change.

Our point estimate of average C0 litter size >2, and observations of females with three C1s (Supplementary Table S4), is interesting because triplet litters are thought to be rare in most polar bear subpopulations except for Western Hudson Bay^53^. Higher litter size may reflect maternal body condition prior to entering the den^54,55^ and sufficient post-denning prey availability to support larger family groups^25,52^. However, the CS region is experiencing high rates of sea-ice loss^17^. During the period 1979–2014, the open water period increased by 80 days^56^ and large declines in summer sea-ice extent are projected to continue^33^. Although several studies (including this one) have suggested that the CS polar bear subpopulation has not been negatively affected by ice loss to date, telemetry data indicate that twice as many collared females are spending the summer on shore, and are remaining there 30 days longer, compared to two decades ago^36^. As sea-ice loss continues it is uncertain how much additional time polar bears can spend in poor foraging habitats (e.g., land, sea ice over less-productive waters of the polar basin)^57,58^ without experiencing negative nutritional and demographic effects. The availability of supplemental nutrition in the form of stranded carcasses of gray whales (*Eschrichtius robustus*), bowhead whales (*Balaena mysticetus*), and other marine mammals along the coastlines of the CS region may become increasingly important as sea-ice loss continues, although these resources are unlikely to compensate for the effects of sea-ice loss in the long term^59^.

### Density and Abundance

Estimates of density and abundance from the current study appear broadly consistent with previous estimates for the CS subpopulation, although methodological differences preclude direct comparisons. Estimated density within the core sampling area (${\bar{D}}_{sampling}$) was 0.0030 bears/km^2^ (0.0016–0.0060). This is similar to a spring on-ice density of 0.0031 bears/km^2^ (SE = 0.0019) estimated using distance-sampling methods in 1987 for the area around Cape Lisburne, Alaska, which spatially overlaps our study area (Fig. 1)^60^. Our estimated abundance within the CS subpopulation boundary (2,937) is within the range of 2,000–5,000 bears that Belikov 1992^23^ suggested would be necessary to explain the number of females observed denning on Wrangel Island in the 1970s and 1980s, noting that the spring on-ice area to which this previous estimate applied was not defined. Based on an area of 815,000 km^2^ (excluding land) within the CS subpopulation boundary, 2,937 bears correspond to an average density of 0.0036 bears/km^2^ (0.0019–0.0073). This is similar to an average spring on-ice density of 0.0041 bears/km^2^ (SE = 0.0026) obtained from a meta-analysis of 12 polar bear subpopulations in Canada^10^. Evans *et al*.^61^ used distance sampling to estimate an autumn on-ice density of 0.0068 bears/km^2^ (95% CI = 0.0032–0.0140) for portions of the Chukchi and Beaufort seas overlapping the northeastern corner of the CS subpopulation boundary. As expected, that value is higher than our estimate because sampling in Evans *et al*.^61^ occurred during the ice-retreat season when polar bears concentrate along the ice edge.

The need to estimate abundance for a management area or population range that is larger than the region where sampling occurs is a common challenge in wildlife research and management. Our approach of extrapolating local densities based on habitat metrics has been used in other studies^30,62^, and is supported by a recent meta-analysis suggesting positive relationships between indices of habitat selection and abundance^44^. The CS polar bear subpopulation is likely to meet key assumptions of density extrapolation, including that a population is relatively stable and at equilibrium with its environment^63^. First, polar bears are long-lived animals characterized by high adult survival, and thus unlikely to exhibit large annual fluctuations in abundance. Although there is evidence for declines due to sea-ice loss in some polar bear subpopulations^14,50^, others are stable or increasing and recent case studies suggests that multiple interacting factors influence when and how the effects of sea-ice loss will occur^26^. Second, harvest mortality was likely relatively low and well-distributed throughout the region^45,46^, precluding human-caused source-sink dynamics. Third, our habitat-quality metric was based on contemporary data and included functional responses in habitat selection patterns^29^. Nonetheless, estimates of abundance from the current study should be interpreted with caution. Temporal and individual heterogeneity in recapture probabilities, discussed above and not accounted for in our model, are common sources of bias in estimates of abundance^31^. Also, information on the density and distribution of prey species was not available for inclusion as covariates in the resource selection functions underlying our habitat-quality metric^29^. Polar bears collared in the study area west of Alaska moved throughout the entire region, but few locations were available in the far western portion of the CS subpopulation boundary in the spring (e.g., <1% of bear locations in March and April occurred west of Wrangel Island). As a result, our analyses applied to the CS subpopulation boundary^26^ and we did not estimate abundance within a larger reference area for CS polar bears as defined under the *Agreement between the Government of the United States of America and the Government of the Russian Federation on the Conservation and Management of the Alaska-Chukotka Polar Bear Population* (U.S.-Russia Agreement; United States T. Doc. 107–10), a bilateral treaty signed in 2000. Although circumpolar resource selection functions suggest that sea ice over the continental shelf north of Siberia includes high-value polar bear habitats^64^, the area under the U.S.-Russia Agreement extends west into the Eastern Siberian Sea, which is characterized by lower *in situ* primary production^65^ that could influence species abundance at higher trophic levels.

### Integrated Population Model

Integrated population models offer several advantages including improved precision, reduced bias, estimation of otherwise confounded parameters, and the ability to use all available data to make the best possible inference on a study population^66^. To date, many IPMs have focused on populations with relatively simple life histories (e.g., only juvenile and adult stages)^67^. Here we demonstrate the usefulness of IPMs for mobile, long-lived species by incorporating a complex multistate projection model that reflects sex-specific life history characteristics and explicitly models movement patterns. Our model allowed for extended maternal care, variable reproductive intervals, hidden or partially observable states, sex-specific projection matrices that share information, estimation of parameters that lack direct data (e.g., C0 litter size), and integration of capture-recapture and satellite telemetry data. The model did not meet the assumption of independent datasets previously specified for IPMs^27^, because capture data were used to estimate vital rates in the multievent portion of the model, and to estimate abundance in the count portion of the model. However, simulations suggest that violation of the independence assumption has relatively minor effects on estimates of sampling variance^68^, while IPMs offer clear benefits in terms of precision and accuracy. Additional work is needed to better understand effects of violation of the independence assumption for species with complex life histories.

We used quantitative and qualitative information from the scientific literature and Traditional Ecological Knowledge (TEK) to establish informative Bayesian priors on some parameters. This can be beneficial when data are limited. For instance, breeding probability of adult females with C0s or C1s (*B*_2_) was weakly identifiable because it is a rare event for CS polar bears. As such, we included an informative prior based on previously published estimates^35^, which improved parameter identifiability. Similarly, it can be advantageous to utilize prior information for population processes that are well documented. Polar bear survival has been estimated from multiple demographic case studies (Supplementary Table S3)^26^. In developing our informative priors we assumed that these estimates were approximately unbiased and were informative about survival of CS bears (Supplementary Methods). This was justified by ecological studies^24,25^ and TEK^69^ indicating that CS bears have exhibited good nutritional condition and reproduction in recent years, which suggests that survival has not been depressed through density-dependent mechanisms^70^; and by the fact that human-caused removal rates were within the typical range for the species and we were not aware of other, atypical density-independent sources of mortality. Because our priors were based on survival estimates for subpopulations with variable demographic status and across a wide geographic range^26^, they did not correspond to a specific demographic status (e.g., a negative or positive population growth rate) but rather represented empirical evidence for the range of survival rates exhibited by the global population of polar bears in recent decades. Although survival probabilities were estimable from the data alone, use of informative priors reduced the lower range of posterior distributions, which sometimes were implausible for a long-lived mammal^71^ when vague priors were used (Supplementary Fig. S3). Estimates of other vital rates and subpopulation abundance were robust to the choice of priors on survival (Supplementary Methods).

The IPM framework is sufficiently flexible to accommodate changes to the sampling and analytical design that may improve parameter estimates and allow investigation of temporal variation. Extending our approach to an integrated spatially explicit capture-recapture framework^72^ should be an area of continued research, as it may better address the interconnection between animal movements, the dynamic nature of sea ice, detection probabilities, and demographic processes. Other potential future directions include: (i) increased sample size and expanded geographic distribution of sampling effort; (ii) using re-sightings of individuals within a sampling season to develop a robust design, providing more reliable estimates of survival and movements^73^; (iii) developing telemetry tags that have a low failure rate, are worn for multiple years, and can be applied to all sex and age classes; and (iv) extrapolating densities using a habitat metric that reflects the distribution of prey, or integrating additional count data for the target species, to improve abundance estimates.

### Summary

We developed a model that builds upon previous findings^24,29^, incorporates prior information, and integrates multiple data types to estimate vital rates and provide the first empirical estimates of abundance for the CS polar bear subpopulation. Our analysis provides information that is urgently needed for conservation planning^15^ and co-management of subsistence harvest by federal and Native partners under the U.S.-Russia Agreement^74^. It also identifies study design considerations to help reduce future analytical assumptions, uncertainties, and potential biases. The methods presented here are broadly applicable to researchers interested in IPMs, particularly for large carnivores and other species that present similar research challenges.

## Electronic supplementary material

Supplementary Materials

## Data Availability

Data for the CS polar bear subpopulation and computer code for the IPM^75^ are available in the Dryad Digital Repository repository: 10.5061/dryad.692jb15.
