# Supplementary material for: Integrated Population Modeling Provides the First Empirical Estimates of Vital Rates and Abundance for Polar Bears in the Chukchi Sea
Source: Sci Rep. 2018 Nov 14;8:16780. doi: 10.1038/s41598-018-34824-7 (PMC6235872; doi:10.1038/s41598-018-34824-7)
Supplement: Supplementary file 1 — Supplementary Materials [file 41598_2018_34824_MOESM1_ESM.pdf]

Integrated Population Modeling Provides the First Empirical Estimates of Vital Rates and Abundance for Polar Bears in the Chukchi Sea. Regehr, E.V., Hostetter, N.J., Wilson, R.R., Rode, K.D., St. Martin, M. & Converse, S.J. *Scientific Reports*, DOI:10.1038/s41598-018-34824-7 (2018).

Supplementary Methods

Study Area and Data Collection

Polar bears were physically captured on the sea ice west of Alaska (Fig. 1) in 2008-2011, 2013, and 2015-2016. During the spring sampling period, all sighted polar bears were immobilized, when safe to do so, using projectile syringes containing zolazepam-tiletamine (Telazol®)[1]. Captured bears were marked with plastic ear tags and lip tattoos for individual identification, and sex was determined. Limits of the study area were defined by the operational range of research aircraft (Fig. 1). Each year, we attempted to search the entire study area although annual search patterns varied as a function of weather, sea-ice conditions, and other factors. Helicopter search effort during capture operations was approximately 88, 107, 88, 117, 103, 111, and 111 hours in 2008-2011, 2013, and 2015-2016, respectively.

We defined the multiyear core sampling area by calculating daily Brownian bridge utilization distributions (UDs)[2] from global positioning system (GPS) track log locations for the helicopter used in capture operations, using the *BBMM* package in the R programming language[3]. Each daily UD was scaled to the range [0,1], after which the daily UDs were summed and rescaled. The core sampling area was defined as the 95% probability contour of the resulting map (Fig. 1). This approach delineated, in a reproducible manner, the portion of the study area that was consistently searched for polar bears in each year that sampling occurred.

We defined the southern portion of the Chukchi Sea (CS) subpopulation boundary as the extent of a detailed hull (XTools Pro v. 9.2) calculated from location data for collared adult female bears from 2008-2016, using the methods of Wilson *et al.*[4]. This excluded regions not used by polar bears during our study period, resulting in an area (excluding land) of approximately 815,000 km^2 within the CS subpopulation boundary. The CS subpopulation boundary is encompassed by the area specified under a bilateral treaty entitled the *Agreement between the Government of the United States of America and the Government of the Russian Federation on the Conservation and Management of the Alaska-Chukotka Polar Bear Population* (United States T. Doc. 107-10). In this paper, we estimated abundance for the area within the CS subpopulation boundary only, not for the larger area defined under this treaty.

For collared bears, transmission intervals varied from every 1-3 days for satellite collars and every 1-4 hours for GPS collars[4,5]. Collars were set to automatically drop off approximately 14 months after deployment. Some collars stopped transmitting prematurely due to electronic or physical failure (e.g., tag loss). Young bears were not collared to avoid injury during growth, and adult males were not collared because their large necks prevent collar retention. To standardize telemetry data for analysis, we used the continuous-time correlated random walk model described in Johnson *et al*.[6] and implemented in the *crawl* package[7] in R to predict daily locations for data collected < 7 days apart, following the methods of Rode *et al*.[8]. For each adult female that was given a collar in year *t* and did not experience collar failure, we derived a spring use area as the 95% probability contour of a Brownian bridge UD[3] calculated with location data from 15 March to 01 May of year $t + 1$. A bear was considered in the study area in year $t + 1$ if its spring use area exhibited > 0 proportional overlap with the core sampling area[9]. We also applied a control-chart algorithm to collar temperature data[10] to identify denning events, following the methods of Rode et *al*.[8]. Because only parturient females enter maternal dens[11], a bear that emerged from a den in year $t + 1$ could either have no cubs or have a litter of age-zero cubs (C0), depending on whether ≥ 1 cub was born and survived to the sampling period. This information on the reproductive status of collared bears was used to define observation events in the multievent capture-recapture data.

Polar bear movements in the CS region are strongly influenced by the availability of sea ice, which bears use as a platform for hunting[4]. During the summer and autumn some bears come onto land while others remain on the ice as it recedes toward the polar basin. Wrangel and Herald islands, in Russia, are particularly important as maternal denning habitat and terrestrial refuges during the ice-retreat season[8].

Integrated Population Model

In the integrated population model (IPM), transitions among states in the population projection models (Fig. 2, Fig. 3) were defined as occurring between the spring sampling periods in year *t* and $t + 1$. Unless otherwise noted, parameters in the IPM were constrained to be time constant and not vary as a function of temporary emigration status, to limit the number of

parameters. We assumed that movement probabilities for females not transitioning into the state with age-zero cubs (AFC0; probabilities ψ_1^{II} and ψ_1^{OO}) were the same as for males, due to the lack of telemetry data for male bears. Bears entered the life cycle matrix at age 2 years. We assumed an equal sex ratio for age-one cubs, thus the recruitment rate for age-two males (M2Y) was modeled as one-half the number of age-one cubs (C1) that survived to age two (C2), as determined by reproductive parameters in the female life cycle matrix (Fig. 3).

Reproductive Count Data

Following the methods of Hunter *et al.*[12] and Regehr *et al.*[13], the parameters of the multinomial ($\omega_{L1[1:3]}$) describing the probability that an adult female with age-one cubs (AFC1) has 1, 2, or 3 C1s, were modeled as a function of C0 litter size probabilities ($\omega_{L0[1:3]}$) and C0 survival (ϕ_{C0}), conditional on at least one C0 cub surviving (ϕ_{L0}). Specifically,

$$\omega_{L1[1]} = \begin{pmatrix} (\omega_{L0[1]}\phi_{C0}) + 2\left(\omega_{L0[2]}\phi_{C0}(1-\phi_{C0})\right) \\ +3(\omega_{L0[2]}\phi_{C0}(1-\phi_{C0})^2) \end{pmatrix} / \phi_{L0}, \quad (1)$$

$$\omega_{L1[2]} = \left((\omega_{L0[2]}{\phi_{C0}}^2) + 3(\omega_{L0[3]}{\phi_{C0}}^2(1-\phi_{C0}))\right)/\phi_{L0}, \text{ and} \quad (2)$$

$$\omega_{L1[3]} = (\omega_{L0[3]}{\phi_{C0}}^3)/\phi_{L0}. \quad (3)$$

Expected C0 and C1 litter size (l_{L0} and l_{L1}, respectively) were then derived parameters:

$$l_{L0} = \omega_{L0[1]} + 2\omega_{L0[2]} + 3\omega_{L0[3]}, \text{ and} \quad (4)$$

$$l_{L1} = \omega_{L1[1]} + 2\omega_{L1[2]} + 3\omega_{L1[3]}. \quad (5)$$

To directly link these processes to the female life cycle matrix, C0 and C1 litter survival probabilities are written as functions of litter size and cub survival[13]:

$$\phi_{L0} = 1 - \begin{pmatrix} (1-\phi_{C0})\omega_{L0[1]} + (1-\phi_{C0})^2\omega_{L0[2]} \\ +(1-\phi_{C0})^3\omega_{L0[3]} \end{pmatrix}, \text{ and} \quad (6)$$

$$\phi_{L1} = 1 - ((1-\phi_{C1})\omega_{L1[1]} + (1-\phi_{C1})^2\omega_{L1[2]} + (1-\phi_{C1})^3\omega_{L1[3]}). \quad (7)$$

This approach permitted estimation of C0 litter size despite the near-absence of AFC0s in the study area.

Polar bears are generally weaned (i.e., separated from their mothers) as C2s in the spring[14]. Because the timing of weaning overlapped with our sampling period, we captured both dependent (i.e., with their mothers) and independent C2s. We estimated the probability

that a C2 was weaned prior to our sampling period (W), conditional on survival, from counts of independent C2s ($n_{weanC\ \ ,t}$) and the total number of C2s observed ($n_{C2,t}$), assuming a binomial distribution:

$$n_{weanC2,t} \sim Binomial(W, n_{C2,t}). \quad (8)$$

Estimating weaning probability was necessary for accurate estimation of recruitment (e.g., to account for adult females without dependent young [AFNC] in year t, which had been AFC1s in year t – 1 and successfully weaned their cubs).

Multievent Capture-Recapture Data

The observation component of the IPM used a multievent structure[15] that included both direct and telemetry observation events. Direct observations were based on physical captures during which a bear's location, sex, age, and reproductive status were known, allowing assignment to a specific state. In contrast, some telemetry observations were based on remote data only and therefore associated with state uncertainty (e.g., a collared adult female was known to be alive, out of the study area, and to have denned, but reproductive state was uncertain because whether the bear was in state AFNC or AFC0 after denning depended on whether reproduction was successful, which could not be determined from telemetry data; Supplementary Table S2, Supplementary Figure S2). We modeled observation data of individual i in year t, $y_{i,t}$, as a function of the state of individual i in year t as well as individual- and time-specific factors (e.g., presence of a functional collar). We assumed $y_{i,t}$ is a categorical random variable:

$$y_{i,t} | z_{i,t} \sim Cat(\Pi_{i,t,z_{i,t}}^{sex[i]}), \quad (9)$$

where $\Pi_{i,t,z_{i,t}}^{sex[i]}$ is the sex-specific vector of detection probabilities for individual i in year t. For males, only direct observation data were available. Males inside the study area were detected with probability p, and males outside the study area were detected with probability 0 (i.e., could not be observed).

For female polar bears, detection data in the IPM were coded as 17 event types corresponding to direct and telemetry observation events, some of which included state uncertainty (Supplementary Table S2). The female observation event matrix, which describes

the relationships between observation events and life cycle states, is provided in Supplementary Figure S2. We used a binary indicator variable, $R_{i,t}$, to denote if female i was released with a collar ($R_{i,t} = 1$), or without a collar ($R_{i,t} = 0$), in year t. Due to the possibility of collar loss or failure, a female alive in year $t + 1$ may not have a functional collar due to not having received a collar in year t, or having received a collar that failed prior to sampling in year $t + 1$. We directly estimated collar failure probability (γ_t) since all bears also were individually-marked with lip tattoos (i.e., double-tagging methods)[16]. We modeled γ_t as time dependent because several research studies using the same collars reported variable performance over the years 2008-2016 (U.S. Fish and Wildlife Service *unpublished data*). We assumed that the probability of observing an adult female with a functional collar was 1.0, regardless of whether the bear was in or out of the study area. Remote telemetry observations provided only partial information on an individual's state unless it was also physically recaptured. State uncertainty in remote telemetry observations was addressed through event assignment parameters, $\alpha_{s,j}$, that estimated the probability that a bear in state s was observed with event j given that it was encountered (Supplementary Figure S2)[15].

Abundance Estimation

When fitting the IPM, some outside state abundances were confounded in the first and second years, 2008 and 2009, due to a lack of count data for outside states. To remove this confounding we assumed a first-year stage distribution (F) that described the distribution of age-classes across the entire population. Specifically, we used F = (0.05, 0.04, 0.15, 0.13, 0.09, 0.07, 0.05, 0.04, 0.03, 0.35) for states F2Y, F3Y, AFNC, AFC0, AFC1, AFC2, M2Y, M3Y, M4Y, and AM, respectively. This is the stable-stage distribution for a hypothetical population with an average capacity for growth as defined in Regehr *et al.*[13], which we assumed to be broadly representative of polar bear populations at equilibrium. To evaluate sensitivity to F, we explored two additional first-year, stable-stage distributions corresponding to hypothetical polar bear populations with low and high capacity for growth[13], and found that posterior estimates of key parameters were nearly identical. The following equations describe how first-

year state-specific abundances were derived for inside and outside states. First, we assumed first-year abundances for inside states were independent Poisson random variables:

$$N_{s[in],1} \sim \text{Poisson}(\lambda_{s[\text{in}]}), \quad (10)$$

where $s[\text{in}]$ denotes all inside states. Adult males were used as the reference starting population, and thus the number of adult males outside the study area ($N_{\text{AM[out]},1}$) was also assumed to be a Poisson random variable:

$$N_{\text{AM[out]},1} \sim \text{Poisson}(\lambda_{\text{AM[out]}}), \quad (11)$$

where the total number of adult males in year 1 is the sum of animals located inside and outside the study area:

$$N_{\text{AM[total]},1} = N_{\text{AM[in]},1} + N_{\text{AM[out]},1}. \quad (12)$$

First-year abundances for all other age-classes where then modeled as Poisson random variables, with an expected rate relative to $N_{\text{AM[total]},1}$ and F:

$$N_{s[total],1} \sim \text{Poisson}\left(N_{\text{AM[total]},1}\left(\frac{F_s}{F_{AM}}\right)\right), \quad (13)$$

where F_s/F_{AM} is the proportion of the population in stage s relative to the proportion of adult males (i.e., individuals in stage AM). Finally, first-year abundances for outside states ($N_{s[outside],1}$) were derived as the difference between first year inside and total abundances,

$$N_{s[outside],1} = N_{s[tota\ ],1} - N_{s[inside],1}. \quad (14)$$

For $t > 1$, state-specific abundances were functions of transition probabilities in the life cycle matrix (Fig. 2 and Fig. 3) and abundances in the previous years. Our approach provides a flexible framework to model partially observable or unobservable states in IPMs, where F informs the first-year stage distribution but does not affect first-year total abundance nor restrict stage distributions after the first year. Abundances for the population of bears outside the study area were also estimated as part of the IPM, however these values are not reported since the spatial domain of this outside population is unknown and biological interpretations are not possible.

Model Implementation

Bayesian Priors

We used vague $\mathrm{Unif}(0,1)$ priors for transition (ψ_1^{II}, ψ_1^{OO}, ψ_2^{II}, ψ_2^{OO}), weaning (W), tag failure (γ_t), recapture (p), and cub survival (ϕ_{C1}, ϕ_{C0}) probabilities. $\mathrm{Gamma}(1,1)$ hyperpriors were used to enforce vague $\mathrm{Dirichlet}$ priors for litter size probabilities ($\omega_{L0[1:3]}$, $\omega_{L1[1:3]}$) and event assignment parameters ($\alpha_{s,[1:J_s]}$)[17]. Independent $\mathrm{Gamma}(0.001,0.001)$ priors were used for $\lambda_{s[\mathrm{in}]}$ and $\lambda_{\mathrm{AM[in]}}$ (first-year abundances).

Breeding probability B_1 was given a vague $\mathrm{Unif}(0,1)$ prior distribution. However, preliminary analyses indicated B_2 (breeding probability for females with C0 or C1) was weakly identifiable. We therefore used an informative $\mathrm{Beta}(2.1,11.4)$ prior distribution, which has an approximate mode of 0.10 and 95% credible interval (CRI) of (0.02, 0.38), based on an estimate of this parameter for the adjacent Southern Beaufort Sea polar bear subpopulation[18].

We used informative Beta priors for subadult and adult survival: $\mathrm{Beta}(33.96, 4.20)$, $\mathrm{Beta}(150.43, 11.32)$, $\mathrm{Beta}(11.28, 2.48)$, and $\mathrm{Beta}(33.96, 4.20)$ for ϕ_{SF}, ϕ_{AF}, ϕ_{SM}, and ϕ_{AM}, respectively. These were developed by matching the moments of a Beta distribution to the mean and standard deviation of mean estimates of total survival (i.e., including harvest mortality) from capture-recapture studies for 12 polar bear subpopulations with available data (Supplementary Table S3). Because they were based on survival estimates from case studies for subpopulations with variable demographic status[19] and across a wide geographic range, these priors did not correspond to a specific demographic status (e.g., a negative or positive population growth rate) but rather represented empirical evidence for the range of survival rates exhibited by the global population of polar bears in recent decades[20].

To evaluate sensitivity to the choice of priors, we fit the IPM under three approaches to developing priors and compared the resulting parameter estimates. Approach one used informative priors, as described above and in the main text, based on the mean values of total survival in Supplementary Table S3. Approach two used an alternative set of age- and sex-specific informative priors developed by simulating 5000 values from each of the case studies in Supplementary Table S3, using the reported study-specific means and standard errors. We then matched the moments of a Beta distribution to the mean and standard deviation of the full set

of simulated data. Comparison of the Beta priors resulting from these two approaches demonstrates that approach two resulted in more diffuse distributions with increased supported for lower survival estimates (Supplementary Figure S3). Third, we fit the IPM using vague $\mathrm{Unif}(0,1)$ priors. Results of the sensitivity analysis showed that posterior distributions of survival estimates largely overlapped across the three approaches, with slightly higher mode values for approach one relative to approach two and vague priors (Supplementary Figure S3). To summarize the posterior distributions shown in Supplementary Figure S3, mode estimates of ϕ_{SF}, ϕ_{AF}, ϕ_{SM}, and ϕ_{AM} were approximately 6.8%, 2.3%, 1.4%, and 1.1% higher, respectively, for approach one to developing informative priors compared to approach two. Posterior distributions of estimates for the other vital rates and abundance were robust to choice of priors. For example, the mode estimates and 95% CRIs for breeding probability (B_1) were 0.83 (0.71 – 0.90) and 0.83 (0.71 – 0.90) for approaches one and two to developing informative priors, respectively (i.e., values were the same, at the level of precision we report). The mode estimates and 95% CRIs for study area abundance ($\bar{N}_{study}$) were 296 (176 – 513) and 295 (181 – 486) for approaches one and two to developing informative priors, respectively.

Parameter Restrictions

We restricted $N_{\mathrm{AM[out]},t} \geq N_{\mathrm{AM[in]},t}$ to prevent possible parameter switching. This restriction had no impact on the general results, but prevented $N_{\mathrm{AM[out]},t}$ from dropping to implausible values (e.g., zero adult males outside the study area).

Goodness-of-Fit

We evaluated goodness-of-fit (GOF) using Bayesian *p*-values for each component dataset similar to methods described in Besbeas and Morgan[21]. For count data (litter size, weaning, and state-specific counts), we create replicated data sets as part of the IPM and compared the observed and expected values using a χ^2 discrepancy metric[22]. GOF for the multievent model was evaluated outside the IPM using tests for standard Cormack-Jolly-Seber (CJS) models, as GOF tests for multievent models are not yet available[23]. First, we simplified the capture-recapture dataset to only include direct observations (coded 1) and non-observations

(coded 0). Second, we calculated Bayesian *p*-values using a Freeman-Tukey fit statistics[24] for a fully constrained CJS model where survival and recapture probabilities were time constant, and a global model where survival and recapture probabilities varied by year[23].

We deemed the overall fit of the IPM sufficient. There was no evidence of lack of fit in the capture-recapture model component (p = 0.31 and 0.21 for the fully constrained and global CJS models, respectively) or litter size model component (p = 0.39), and GOF for state-specific count data was marginal (p = 0.09). Marginal fit for the count data was likely due to un-modeled heterogeneity in recapture probabilities, emphasizing the need for caution when interpreting estimates of survival and abundance from this study. The Bayesian *p*-value for weaning data was low (p = 0.03), which was likely due to un-modeled interannual variation (Supplementary Table S5). We did not include additional model complexity to address this possible lack of fit as weaning probability was a relatively minor parameter in the IPM that served primarily to account for temporal overlap between sampling occasions and the period when C2s separate from their mothers.

We fit the IPM running three parallel Markov Chain Monte Carlo (MCMC) simulations. Each chain contained 10,000 adaptive, 20,000 burn-in, and 1,500,000 posterior iterations thinned by 100 to reduce file size, for a total of 45,000 saved posterior iterations. Chain convergence was visually evaluated and verified using the Gelman-Rubin statistic ($\hat{R}$)[25]. Results are presented as posterior modes and 95% CRIs.

Density Extrapolation

Estimates of abundance within the CS subpopulation boundary as described in the main text ($\bar{N}^{*}_{CS}$) were derived by extrapolating densities from the study area and did not include adult females with age-zero cubs (AFC0) and age-zero cubs (C0). To account for this, we adjusted the extrapolated estimates of abundance as follows:

$$\bar{N}_{CS} = \bar{N}^{*}_{CS} \times (1 + r_{AFC0} + r_{AFC0} \times l_{L0}) , \qquad (15)$$

where $\bar{N}_{CS}$ is the total number of bears (i.e., including AFC0 and C0) within the CS subpopulation boundary; r_{AFC0} is a ratio representing the expected number of bears in state AFC0 divided by $\bar{N}^{*}_{CS}$; and l_{L0} is the previously-described mean litter size for C0. We estimated

r_{AFC0} from stable stage distributions calculated from the estimated vital rates using a matrix projection model[26]. Density extrapolation was implemented using a bootstrap procedure that drew random samples, with replacement, from the sampling distribution for the proportion of time that individuals spent within the sampling area during the sampling period ($\hat{q}$), and the MCMC posterior chains for the habitat-quality metric (h_x)[5]; and correlated random samples from the MCMC posterior chains for $\overline{N}_{study}$, l_{L0}, and the vital rates used to estimate r_{AFC0}. This produced estimates of abundance that accounted for the covariance structure of estimated parameters.

REFERENCES

1 Stirling, I., Spencer, C. & Andriashek, D. Immobilization of polar bears (*Ursus maritimus*) with Telazol® in the Canadian Arctic. *J Wildl Dis* **25**, 159-168 (1989).

2 Horne, J. S., Garton, E. O., Krone, S. M. & Lewis, J. S. Analyzing animal movements using Brownian bridges. *Ecology* **88**, 2354-2363 (2007).

3 Nielson, R. M., Sawyer, H. & McDonald, T. L. BBMM: Brownian bridge movement model. *R package version 3.0. https://CRAN.R-project.org/package=BBMM* (2013).

4 Wilson, R. R., Horne, J. S., Rode, K. D., Regehr, E. V. & Durner, G. M. Identifying polar bear resource selection patterns to inform offshore development in a dynamic and changing Arctic. *Ecosphere* **5**, 24, doi:10.1890/es14-00193.1 (2014).

5 Wilson, R. R., Regehr, E. V., Rode, K. D. & St. Martin, M. Invariant polar bear habitat selection during a period of sea ice loss. *Proc R Soc Biol Sci Ser B* **283**, 20160380, doi:10.1098/rspb.2016.0380 (2016).

6 Johnson, D. S., London, J. M., Lea, M.-A. & Durban, J. W. Continuous-time correlated random walk model for animal telemetry data. *Ecology* **89**, 1208-1215 (2008).

7 Johnson, D. S. Crawl: fit continuous-time correlated random walk models to animal movement data. *R package version 3.1.4. http://cran.r-project.org/package=crawl* (2013).

8 Rode, K. D. *et al.* Increased land use by Chukchi Sea polar bears in relation to changing sea ice conditions. *PLoS ONE*, 10.1371/journal.pone.0142213 (2015).

9 Fieberg, J. & Kochanny, C. O. Quantifying home-range overlap: The importance of the utilization distribution. *J Wildl Manage* **69**, 1346-1359 (2005).

10 Olson, J. W. *et al.* Collar temperature sensor data reveal long-term patterns in southern Beaufort Sea polar bear den distribution on pack ice and land. *Mar Ecol Prog Ser* **564**, 211-224 (2017).

11 Ramsay, M. A. & Stirling, I. Reproductive biology and ecology of female polar bears (*Ursus maritimus*). *J Zool (Lond.)* **214**, 601-634 (1988).

12 Hunter, C. M. *et al.* Climate change threatens polar bear populations: a stochastic demographic analysis. *Ecology* **91**, 2883-2897 (2010).

13 Regehr, E. V., Wilson, R. R., Rode, K. D., Runge, M. C. & Stern, H. Harvesting wildlife affected by climate change: a modeling and management approach for polar bears. *J Appl Ecol* **54**, 1534-1543 (2017).

14 Amstrup, S. C. Polar Bear (*Ursus maritimus*) in *Mammals of North America: Biology, Management, and Conservation* (eds. George A. Feldhamer, Bruce C. Thompson, & Joseph A. Chapman) 587-610 (John Hopkins University Press, 2003).
15 Pradel, R. Multievent: An extension of multistate capture-recapture models to uncertain states. *Biometrics* **61**, 442-447 (2005).
16 Reynolds, T. J. *et al.* Integrated data analysis in the presence of emigration and mark loss. *J Agric Biol Environ Stat* **14**, 411-431 (2009).
17 Royle, J. A. & Dorazio, R. M. *Hierarchical Modeling and Inference in Ecology: The Analysis of Data from Populations, Metapopulations, and Communities*. (Academic Press, 2008).
18 Regehr, E. V., Hunter, C. M., Caswell, H., Amstrup, S. C. & Stirling, I. Survival and breeding of polar bears in the southern Beaufort Sea in relation to sea ice. *J Anim Ecol* **79**, 117-127 (2010).
19 Durner, G. M., Laidre, K. L. & York, G. S. *Polar Bears: Proceedings of the 18th Working Meeting of the IUCN/SSC Polar Bear Specialist Group, 7-11 June 2016, Anchorage, Alaska. Gland, Switzerland and Cambridge, UK: IUCN. xxx + 207pp.* (2018).
20 Eberhardt, L. L. Survival rates required to sustain bear populations. *J Wildl Manage* **54**, 587-590 (1990).
21 Besbeas, P. & Morgan, B. J. T. Goodness-of-fit of integrated population models using calibrated simulation. *Methods Ecol. Evol.* **5**, 1373-1382 (2014).
22 Kéry, M. & Royle, J. A. *Applied Hierarchical Modeling in Ecology: Analysis of distribution, abundance and species richness in R and BUGS: Volume 1: Prelude and Static Models*. First edition (Academic Press, 2016).
23 Lagrange, P., Pradel, R., Belisle, M. & Gimenez, O. Estimating dispersal among numerous sites using capture-recapture data. *Ecology* **95**, 2316-2323 (2014).
24 Brooks, S. P., Catchpole, E. A., Morgan, B. J. T. & Barry, S. C. On the Bayesian analysis of ring-recovery data. *Biometrics* **56**, 951-956 (2000).
25 Gelman, A., Carlin, J. B., Stern, H. S. & Rubin, D. B. *Bayesian Data Analysis*. Second Edition (CRC Press, 2004).
26 Caswell, H. *Matrix Population Models*. Second Edition (Sinauer Associates Inc., 2001).

Supplementary Results

Detection Probabilities

The integrated population model estimated that the probability of physical recapture conditional on being in the study area (p) was 0.17 (0.12 – 0.34). Telemetry event assignment parameter modes ranged from 0.01 to 0.97 (Supplementary Table S6) and often had high levels of imprecision, indicating that telemetry data provided important information that a female was alive and in or out of the study area, but limited information on reproductive state. The probability of telemetry tag failure (i.e., that a collar would stop transmitting prematurely) ranged from 0.11 (0.02 – 0.41) in 2009 to 0.42 (0.23 – 0.67) in 2013 (Supplementary Table S6).

Supplementary Table S1. Parameter, data, and indexing definitions for an integrated population model for polar bears in the Chukchi Sea. AFC0 is the life cycle state for an adult female (AF) with at least one age-zero cub (C0).

Parameter	Definition
ϕ_{AF}	Adult female (≥ 4 years) survival probability
ϕ_{SF}	Subadult female (2-3 years) survival probability
ϕ_{AM}	Adult male (≥ 5 years) survival probability
ϕ_{SM}	Subadult male (2-4 years) survival probability
ϕ_{L0}	Age-zero litter survival probability (i.e., at least one cub survives), conditional on adult female survival
ϕ_{L1}	Age-one litter survival probability (i.e., at least one cub survives), conditional on adult female survival
ϕ_{C0}	Age-zero cub (C0) survival probability, conditional on adult female survival
ϕ_{C1}	Age-one cub (C1) survival probability, conditional on adult female survival
ψ_1^{II}	The probability an individual inside the study area at year t is in the study area at year $t+1$, conditional on survival and not transitioning into state AFC0
ψ_1^{OO}	The probability an individual outside the study area at year t is outside the study area at year $t+1$, conditional on survival and not transitioning into state AFC0
ψ_2^{II}	The probability an individual inside the study area at year t is in the study area at year $t+1$, conditional on survival and transitioning into state AFC0
ψ_2^{OO}	The probability an individual outside the study area at year t is outside the study area at year $t+1$, conditional on survival and transitioning into state AFC0
B_1	Conditional on survival, the probability that a female without cubs or a female with age-two cubs at year t will breed and have at least one age-zero cub that survives until year $t+1$
B_2	Conditional on survival, the probability that a female with age-zero or age-one cubs at year t will breed and have at least one age-zero cu that survives until year $t+1$
W	Probability an age-two cub is independent (i.e., separate from its mother) at the time of sampling
$\omega_{L0[1:3]}$	Probability an age-zero litter consists of one, two, or three cubs
$\omega_{L1[1:3]}$	Probability an age-one litter consists of one, two, or three cubs
l_{L0}	Derived average litter size of age-zero litters
l_{L1}	Derived average litter size of age-one litters
$\lambda_{s,1}$	Mean rate of individuals in state s in year 1
$N_{s,t}$	Latent number of individuals in state s at year t
$N_{study,t}$	Total number of individuals that used the study area during the sampling period at year t

$\overline{N}_{study}$	Multiyear average of the total number of individuals that used the study area
p	Probability of physical recapture inside the study area without use of collar location data to locate the animal
γ_t	Probability a collar applied to an animal at year t fails prior to year $t+1$
$\alpha_{s,j}$	The probability an individual with a functional collar in state s is observed with event j given it was encountered
$\overline{D}_{sampling}$	Multiyear, average density (bears/km^2) within the core sampling area
$\overline{D}^*_{sampling}$	Multiyear, average density (bears/km^2) within the core sampling area, excluding bears in state AFC0 (adult female with age-zero cubs) and their dependent age-zero cubs
$\hat{q}$	Average proportion of time that members of the study population spent inside the core sampling area, during the spring sampling period
$A_{sampling}$	Area (km^2) of the core sampling area
h_x	Habitat-quality metric, derived from resource selection functions, for grid cell x
$\overline{N}^*_{CS}$	Abundance within the Chukchi Sea (CS) subpopulation boundary, excluding bears in state AFC0 and their dependent age-zero cubs
$\overline{N}_{CS}$	Total abundance within the CS subpopulation boundary
r_{AFC0}	Ratio of the expected number of bears in state AFC0 divided by $\overline{N}^*_{CS}$

<u>Data</u>

$y_{i,t}$	Observation event of individual i at year t
$n_{s,t}$	Number of individuals in state s captured at year t
$n_{L1[1:3],t}$	Number of observed age-one litters with one, two, and three cubs at year t
$n_{C2,t}$	Number of observed age-two individuals at year t
$n_{weanC2,t}$	Number of observed independent age-two individuals at year t
$R_{i,t}$	Binary indicator, where 1 indicates individual i received a collar at year t, and 0 otherwise
F	Distribution of age-classes across the entire population, used to initialize the integrated population model

<u>Indexing</u>

t	Year; 1, 2,…, T
i	Individual; 1, 2,…, n
s	State; 1, 2,…, S
j	Event type; 1, 2,…, J_s, where J_s is the total number of possible observation events for an individual in state s
x	Grid cell; 1, 2,…, X, where $X_{sampling}$ is the number of grid cells overlaying the core sampling area, and X_{CS} is the number of grid cells overlaying the CS subpopulation boundary

Supplementary Table S2. Description of observation events for an integrated population model for polar bears in the Chukchi Sea. States in the life cycle models (M2Y, M3Y,…) are defined in the main text. Sample size is the total number of events resulting from physical capture-recapture research conducted 2008-2011, 2013, and 2015-2016. AF is an adult female (≥ 4 years). For males, observation events corresponded directly to life cycle states (i.e., there was no state uncertainty). For females, some observation events were associated with state uncertainty because denning status, as determined from telemetry data, did not uniquely determine reproductive status. Relationships between female observation events and life cycle states are defined in Supplementary Figure S2.

Event type	Event code	Sample size	Description
Direct (male)	M2Y	32	Physical recapture of known M2Y
	M3Y	17	Physical recapture of known M3Y
	M4Y	17	Physical recapture of known M4Y
	AM	129	Physical recapture of known AM
Direct (female)	$F2Y_{in}$	28	Physical recapture of known F2Y
	$F3Y_{in}$	9	Physical recapture of known F3Y
	$AFNC_{in}$	45	Physical recapture of known AFNC
	$AFC0_{in}$	3	Physical recapture of known AFC0
	$AFC1_{in}$	41	Physical recapture of known AFC1
	$AFC2_{in}$	19	Physical recapture of known AFC2
Telemetry (female)	T $AFNC_{in}$	1	Telemetry detection of known AFNC, confirmed through recapture or sighting
	T $AFC0_{in}$	0	Telemetry detection of known AFC0, confirmed through recapture or sighting
	T $AFC1_{in}$	0	Telemetry detection of known AFC1, confirmed through recapture or sighting
	T $AFC2_{in}$	5	Telemetry detection of known AFC2, confirmed through recapture or sighting

	T AFD_{UNK}	8	Telemetry detection of AF that denned and location during sampling period is unknown (true state is uncertain)
	T AFD_{in}	2	Telemetry detection of AF that denned and is located inside the study area (true state is uncertain)
	T AFD_{out}	16	Telemetry detection of AF that denned and is located outside the study area (true state is uncertain)
	T $AFND_{UNK}$	12	Telemetry detection of AF that did not den and location during sampling period is unknown (true state is uncertain)
	T $AFND_{in}$	8	Telemetry detection of AF that did not den and is located inside the study area (true state is uncertain)
	T $AFND_{out}$	11	Telemetry detection of AF that did not den and is located outside the study area (true state is uncertain)
Not detected	-	-	Not detected

Supplementary Table S3. Estimates of total survival (i.e., including harvest mortality) from capture-recapture studies for polar bears. These values were used to establish informative priors for survival in an integrated population model for polar bears in the Chukchi Sea, as described in the main text and Supplementary Methods. Sex- and age-classes are subadult female (2-3 years; SF), adult female (≥ 4 years; AF), subadult male (2-4 years; SM), and adult male (≥ 5 years; AM).

Subpopulation	SF	AF	SM	AM
Baffin Bay[1]	NA	0.95	NA	0.87
Davis Strait-Central[2]	0.92	0.95	0.89	0.94
Davis Strait-Northern[2]	0.90	0.94	0.87	0.92
Davis Strait-Southern[2]	0.92	0.95	0.89	0.94
Gulf of Boothia[2]	0.90	0.92	0.88	0.92
Kane Basin[1]	0.73	0.95	0.52	0.87
Lancaster Sound[4]	0.88	0.94	0.79	0.89
McClintock Channel[5]	0.90	0.90	0.90	0.88
Northern Beaufort Sea[6]	0.91	0.91	0.83	0.83
Norwegian Bay[4]	0.88	0.94	0.79	0.89
Southern Beaufort Sea[7]	0.92	0.95	0.87	0.93
Southern Hudson Bay[8]	0.92	0.91	0.86	0.86
Viscount Melville[9]	0.91	0.91	0.77	0.77
Western Hudson Bay[10]	0.82	0.94	0.75	0.90

References

1 SWG (Scientific Working Group to the Canada-Greenland Joint Commission on Polar Bear). Re-Assessment of the Baffin Bay and Kane Basin Polar Bear Subpopulations: Final Report to the Canada-Greenland Joint Commission on Polar Bear. 31 July 2016: x + 636 pp (2016).

2 Peacock, E., Taylor, M. K., Laake, J. & Stirling, I. Population ecology of polar bears in Davis Strait, Canada and Greenland. *J Wildl Manage* **77**, 463-476 (2013).

3 Taylor, M. K., Laake, J., McLoughlin, P. D., Cluff, H. D. & Messier, F. Demography and population viability of polar bears in the Gulf of Boothia, Nunavut. *Mar Mamm Sci* **25**, 778-796 (2009).

4 Taylor, M. K., Laake, J., McLoughlin, P. D., Cluff, H. D. & Messier, F. Mark-recapture and stochastic population models for polar bears of the high Arctic. *Arctic* **61**, 143-152 (2008).
5 Taylor, M. K., Laake, J., Mcloughlin, P. D., Cluff, H. D. & Messier, F. Demographic parameters and harvest-explicit population viability analysis for polar bears in M'clintock Channel, Nunavut, Canada. *J Wildl Manage* **70**, 1667-1673 (2006).
6 Stirling, I., McDonald, T. L., Richardson, E. S., Regehr, E. V. & Amstrup, S. C. Polar bear population status in the northern Beaufort Sea, Canada, 1971-2006. *Ecol Appl* **21**, 859-876 (2011).
7 Regehr, E. V., Hunter, C. M., Caswell, H., Amstrup, S. C. & Stirling, I. Survival and breeding of polar bears in the southern Beaufort Sea in relation to sea ice. *J Anim Ecol* **79**, 117-127 (2010).
8 Obbard, M. E., McDonald, T. L., Howe, E. J., Regehr, E. V. & Richardson, E. S. Polar Bear Population Status in Southern Hudson Bay, Canada. *U.S. Geological Survey Administrative Report, Reston, Virginia, USA.*, 32 pp (2007).
9 Taylor, M. K., Laake, J., Cluff, H. D., Ramsay, M. & Messier, F. Managing the risk from hunting for the Viscount Melville Sound polar bear population. *Ursus* **13**, 185-202 (2002).
10 Lunn, N. J. *et al.* Demography of an apex predator at the edge of its range: impacts of changing sea ice on polar bears in Hudson Bay. *Ecol Appl* **26**, 1302-1320 (2016).

Supplementary Table S4. Litter sizes for adult females with age-one cubs (AFC1). Observations were made during physical captures of polar bears in the Chukchi Sea, 2008-2016. No fieldwork was performed in 2012 and 2014. For example, of the 11 litters of age-one cubs (C1s) observed in 2010, three had 1 C1, six had 2 C1s, and two had 3 C1s. The relative proportions of litter sizes in 2010 was 0.27, 0.55, and 0.18 for 1 C1, 2 C1, and 3 C1 litters, respectively.

		Observed litter size (proportion)		
Year	Number of observed AFC1	1 C1	2 C1	3 C1
2009	1	1 (1.00)	0 (0.00)	0 (0.00)
2010	11	3 (0.27)	6 (0.55)	2 (0.18)
2011	6	5 (0.83)	1 (0.17)	0 (0.00)
2013	5	2 (0.40)	2 (0.40)	1 (0.20)
2015	4	1 (0.25)	3 (0.75)	0 (0.00)
2016	12	7 (0.58)	5 (0.42)	0 (0.00)
Total	39	19 (0.49)	17 (0.44)	3 (0.08)

Supplementary Table S5. Weaning status for age-two (C2) cubs. Observations were made during physical captures of polar bears in the Chukchi Sea, 2008-2016. No fieldwork was performed in 2012 and 2014. Weaned C2s are independent age-two cubs observed without an adult female. Total C2s is the number of independent and dependent (i.e., with an adult female) age-two cubs.

Year	Number of weaned C2	Number of total C2	% Weaned
2008	1	2	0.50
2009	1	4	0.25
2010	1	5	0.20
2011	10	22	0.45
2013	5	7	0.71
2015	4	9	0.44
2016	0	12	0.00
Total	22	61	0.36

Supplementary Table S6. Detection parameters from an integrated population model for polar bears in the Chukchi Sea. Values are posterior modes and 95% credible intervals (CRI). Parameters include recapture probability (p), collar failure probabilities (γ), and telemetry event assignment parameters (α). Detailed parameter definitions are provided in the main text and Supplementary Table S1.

Parameter	Mode (95% CRI)
p	0.17 (0.12 – 0.34)
γ_{2008}	0.29 (0.10 – 0.57)
γ_{2009}	0.11 (0.02 – 0.41)
γ_{2010}	0.12 (0.04 – 0.35)
γ_{2011}	0.19 (0.07 – 0.43)
γ_{2012}	NA
γ_{2013}	0.42 (0.23 – 0.67)
γ_{2014}	NA
γ_{2015}	0.41 (0.20 – 0.64)
$\alpha_{AFNCo,1}$	0.08 (0.02 – 0.38)
$\alpha_{AFNCo,2}$	0.02 (0.00 – 0.31)
$\alpha_{AFNCo,3}$	0.14 (0.03 – 0.46)
$\alpha_{AFNCo,4}$	0.04 (0.01 – 0.50)
$\alpha_{AFNCo,5}$	0.35 (0.11 – 0.69)
$\alpha_{AFNCu,1}$	0.01 (0.00 – 0.28)
$\alpha_{AFNCu,2}$	0.09 (0.01 – 0.40)
$\alpha_{AFNCu,3}$	0.02 (0.00 – 0.37)
$\alpha_{AFNCu,4}$	0.67 (0.37 – 0.89)
$\alpha_{AFC0o,1}$	0.05 (0.01 – 0.81)
$\alpha_{AFC0o,2}$	0.06 (0.01 – 0.85)
$\alpha_{AFC0o,3}$	0.14 (0.02 – 0.86)
$\alpha_{AFC0u,1}$	0.31 (0.13 – 0.54)
$\alpha_{AFC0u,2}$	0.69 (0.46 – 0.87)
$\alpha_{AFC1o,1}$	0.05 (0.01 – 0.81)
$\alpha_{AFC1o,2}$	0.20 (0.02 – 0.87)
$\alpha_{AFC1o,3}$	0.05 (0.01 – 0.82)
$\alpha_{AFC1u,1}$	0.91 (0.04 – 0.98)
$\alpha_{AFC1u,2}$	0.09 (0.02 – 0.96)
$\alpha_{AFC2o,1}$	0.48 (0.22 – 0.82)
$\alpha_{AFC2o,2}$	0.02 (0.00 – 0.40)
$\alpha_{AFC2o,3}$	0.37 (0.06 – 0.68)

$\alpha_{AFC2u,1}$	0.36 (0.02 – 0.91)
$\alpha_{AFC2u,2}$	0.64 (0.09 – 0.98)
$\alpha_{DEAD,1}$	0.03 (0.01 – 0.55)
$\alpha_{DEAD,2}$	0.97 (0.45 – 0.99)

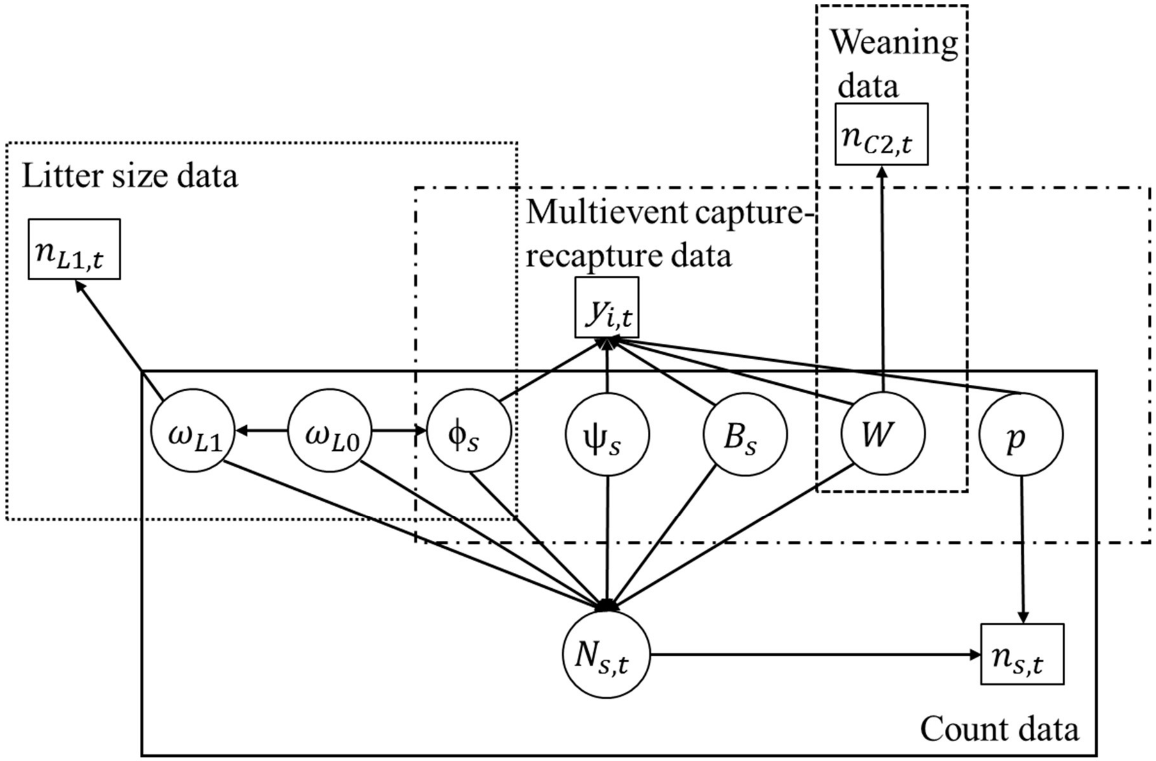

Supplementary Figure S1. Direct acyclic graph of the integrated population model for Chukchi Sea polar bears. Data are represented by rectangles and estimated parameters are represented by circles. Arrows represent dependencies among nodes. For simplicity, different sex- and age-class specific parameters are represented by a single node. A complete list of parameter, data, and indexing definitions is provided in Supplementary Table S1.

	Direct events						Telemetry events			
	$F2Y_{in}$	$F3Y_{in}$	$AFNC_{in}$	$AFC0_{in}$	$AFC1_{in}$	$AFC2_{in}$	T $AFNC_{in}$	T $AFC0_{in}$	T $AFC1_{in}$	T $AFC2_{in}$
$F2Y_{in}$	p	0	0	0	0	0	0	0	0	0
$F2Y_{out}$	0	0	0	0	0	0	0	0	0	0
$F3Y_{in}$	0	p	0	0	0	0	0	0	0	0
$F3Y_{out}$	0	0	0	0	0	0	0	0	0	0
$AFNC_{in}$	0	0	$p(1-R_{i,t-1})+pR_{i,t-1}\gamma_t$	0	0	0	$\alpha_{AFNC\ in,1}R_{i,t-1}(1-\gamma_t)$	0	0	0
$AFNC_{out}$	0	0	0	0	0	0	0	0	0	0
$AFC0_{in}$	0	0	0	$p(1-R_{i,t-1})+pR_{i,t-1}\gamma_t$	0	0	0	$\alpha_{AFC0\ in,1}R_{i,t-1}(1-\gamma_t)$	0	0
$AFC0_{out}$	0	0	0	0	0	0	0	0	0	0
$AFC1_{in}$	0	0	0	0	$p(1-R_{i,t-1})+pR_{i,t-1}\gamma_t$	0	0	0	$\alpha_{AFC1\ in,1}R_{i,t-1}(1-\gamma_t)$	0
$AFC1_{out}$	0	0	0	0	0	0	0	0	0	0
$AFC2_{in}$	0	0	0	0	0	$p(1-R_{i,t-1})+pR_{i,t-1}\gamma_t$	0	0	0	$\alpha_{AFC2\ in,1}R_{i,t-1}(1-\gamma_t)$
$AFC2_{out}$	0	0	0	0	0	0	0	0	0	0
Dead	0	0	0	0	0	0	0	0	0	0

	Telemetry events						
	T AFD_{unk}	T AFD_{in}	T AFD_{out}	T $AFND_{unk}$	T $AFND_{in}$	T $AFND_{out}$	NotDetected
$F2Y_{in}$	0	0	0	0	0	0	$(1-p)$
$F2Y_{out}$	0	0	0	0	0	0	1
$F3Y_{in}$	0	0	0	0	0	0	$(1-p)$
$F3Y_{out}$	0	0	0	0	0	0	1
$AFNC_{in}$	$\alpha_{AFNC\ in,2}R_{i,t-1}(1-\gamma_t)$	$\alpha_{AFNC\ in,3}R_{i,t-1}(1-\gamma_t)$	0	$\alpha_{AFNC\ in,4}R_{i,t-1}(1-\gamma_t)$	$\alpha_{AFNC\ in,5}R_{i,t-1}(1-\gamma_t)$	0	$(1-p)\left((1-R_{i,t-1})+R_{i,t-1}\gamma_t\right)$
$AFNC_{out}$	$\alpha_{AFNC\ out,1}R_{i,t-1}(1-\gamma_t)$	0	$\alpha_{AFNC\ out,2}R_{i,t-1}(1-\gamma_t)$	$\alpha_{AFNC\ out,3}R_{i,t-1}(1-\gamma_t)$	0	$\alpha_{AFNC\ out,4}R_{i,t-1}(1-\gamma_t)$	$(1-R_{i,t-1})+R_{i,t-1}\gamma_t$
$AFC0_{in}$	$\alpha_{AFC0\ in,2}R_{i,t-1}(1-\gamma_t)$	$\alpha_{AFC0\ in,3}R_{i,t-1}(1-\gamma_t)$	0	0	0	0	$(1-p)\left((1-R_{i,t-1})+R_{i,t-1}\gamma_t\right)$
$AFC0_{out}$	$\alpha_{AFC0\ out,1}R_{i,t-1}(1-\gamma_t)$	0	$\alpha_{AFC0\ out,2}R_{i,t-1}(1-\gamma_t)$	0	0	0	$(1-R_{i,t-1})+R_{i,t-1}\gamma_t$
$AFC1_{in}$	0	0	0	$\alpha_{AFC1\ in,2}R_{i,t-1}(1-\gamma_t)$	$\alpha_{AFC1\ in,3}R_{i,t-1}(1-\gamma_t)$	0	$(1-p)\left((1-R_{i,t-1})+R_{i,t-1}\gamma_t\right)$
$AFC1_{out}$	0	0	0	$\alpha_{AFC1\ out,1}R_{i,t-1}(1-\gamma_t)$	0	$\alpha_{AFC1\ out,2}R_{i,t-1}(1-\gamma_t)$	$(1-R_{i,t-1})+R_{i,t-1}\gamma_t$
$AFC2_{in}$	0	0	0	$\alpha_{AFC2\ in,2}R_{i,t-1}(1-\gamma_t)$	$\alpha_{AFC2\ in,3}R_{i,t-1}(1-\gamma_t)$	0	$(1-p)\left((1-R_{i,t-1})+R_{i,t-1}\gamma_t\right)$
$AFC2_{out}$	0	0	0	$\alpha_{AFC2\ out,1}R_{i,t-1}(1-\gamma_t)$	0	$\alpha_{AFC2\ out,2}R_{i,t-1}(1-\gamma_t)$	$(1-R_{i,t-1})+R_{i,t-1}\gamma_t$
Dead	$\alpha_{Dead,1}R_{i,t-1}(1-\gamma_t)$	0	0	$\alpha_{Dead,2}R_{i,t-1}(1-\gamma_t)$	0	0	$(1-R_{i,t-1})+R_{i,t-1}\gamma_t$

Supplementary Figure S2. Female observation event matrix for polar bears in the Chukchi Sea. There are direct observation events (i.e., physical captures) and telemetry observation events. Cell values are detection probabilities in year t for a given observation event (column), given an individual's true state in year t (row). Non-zero cell values indicate the possible observation event(s) corresponding to each life cycle state. States, parameters, and observation events are described in the main text, Supplementary Table S1, and Supplementary Table S2, respectively.

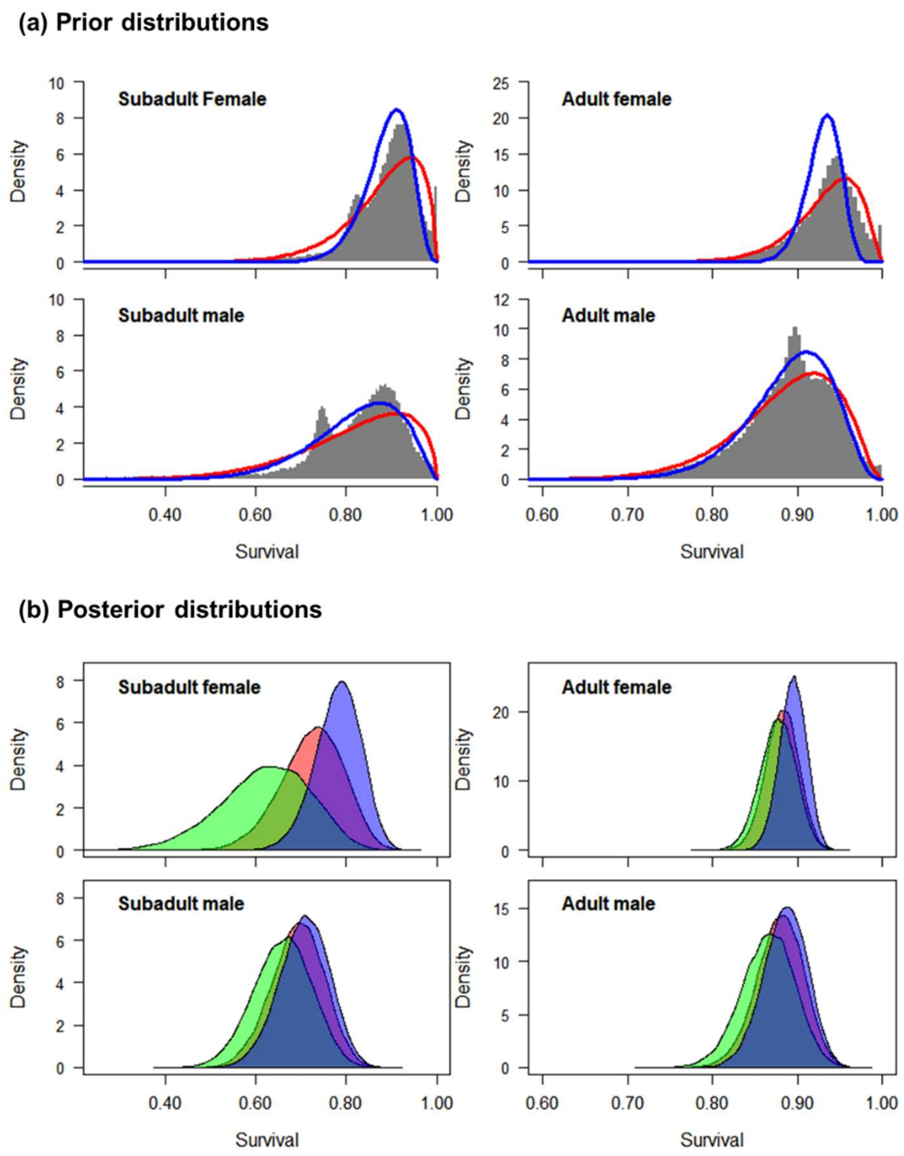

Supplementary Figure S3. Results of a prior sensitivity analysis on survival of independent bears. Panel (a) is density plots of sex- and age-specific Beta prior distributions developed using (1) the mean and standard deviation of the mean across previous polar bear survival studies (blue lines; approach one, as described in the Supplementary Methods), and (2) the mean and standard deviation from simulated sex-, age-, and study-specific survival estimates (grey histograms; n = 5,000 simulated points per study) from the same previous polar bear survival studies (red lines; approach two). Panel (b) is posterior distributions for sex- and age-specific survival from the integrated population model for polar bears in the Chukchi Sea. Posterior

distributions using approach one to developing informative priors are shown in blue, approach two to developing informative priors are shown in red, and vague Uniform(0, 1) priors are shown in green. The three approaches to developing priors are described in the Supplementary Methods.
